# Introduction of Self-Healing and Recyclable Properties into Functionalized Polyisoprene Rubber via Thiol–Ene Reaction

**DOI:** 10.1021/acspolymersau.6c00009

**Published:** 2026-03-06

**Authors:** Yan-Sin Huang, Livy Laysandra, Yu-Cheng Chiu

**Affiliations:** † Department of Chemical Engineering, 562499 National Taiwan University of Science and Technology, No.43, Sec. 4, Keelung Rd., Da’an Dist., Taipei 10607, Taiwan; ‡ Advanced Research Center for Green Materials Science and Technology, 34878 National Taiwan University, Taipei 10617, Taiwan

**Keywords:** polyisoprene, thiol−ene, rubber, stretchable, self-healing, recyclable

## Abstract

Covalently cross-linked rubbers face persistent sustainability challenges due to their irreversible networks hindering recycling, while polarity mismatch complicates the incorporation of additional self-healing materials into vulcanization-free cis-1,4-polyisoprene (PI). To advance the sustainable development of functionalized PI with additional new features while promoting the elasticity and mechanical properties, our group proposes a straightforward one-step free radical-mediated thiol–ene reaction using l-cysteine (LC) as a biodegradable compound that bears three main functional groups consisting of thiol, carboxylic acid, and amine. The thiol group is covalently attached to the PI double bonds via free radical thiol–ene chemistry, while the carboxylic acid and amine groups facilitate noncovalent cross-linking through dynamic hydrogen bonds. As the LC content increases, the functionalized PI-LC-X (with *X* = 10, 30, and 50 denoting the percentage of LC units attached to the PI double bonds) exhibits a synergistic enhancement in the mechanical strength and elasticity. Among them, PI-LC-30 represents the optimal performance in self-healing ability, achieving 100% recovery of toughness at room temperature along with excellent recyclability through acid hydrolysis. This outstanding behavior is attributed to the well-controlled ideal radical thiol–ene reaction (anti-Markovnikov addition), which prevents unwanted chain extension or interchain cross-linking and preserves the linear structure of PI. Maintaining this structural integrity is vital for recyclability, as acid hydrolysis selectively disrupts the reversible hydrogen bonds while keeping the covalent thioether linkages intact, enabling the regeneration of PI-LC-X films with properties closely matching the original material. This strategy effectively addresses polarity mismatch and recyclability challenges, offering a sustainable pathway for functionalizing diene rubbers.

## Graphical abstract

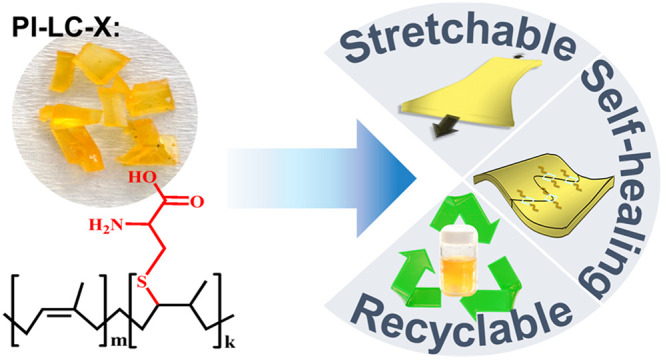

## Introduction

Elastomer modification techniques through various structural alterations, including cross-linking, branching, blending, or molecular weight distribution control, often lead to a sudden growth in innovation driven by opportunities for new high-value functional applications in materials science and engineering.^1–5^ The concept of synthetically cross-linked *cis*-1,4-polyisoprene (PI) is deeply rooted and far-reaching in the field of rubber industry including tires, conveyor belts, gloves, and damping materials.^6–8^ Cross-linked PI as one of the renowned diene elastomeric materials with good fatigue resistance, strong adhesion, and excellent barrier properties against oxygen and water has tempted researchers to expand the utilization of PI into electrical insulators, sensors, medical/healthcare devices, and even next-generation smart devices.^1,3,9,10^ The traditional cross-linking PI generally revolves around the formation of covalent bonds by reacting the carbon–carbon double bonds present in the polymer backbone.^11–13^ The sulfur vulcanization process is the most commonly used technique in the rubber industry through the reaction of PI double bonds with a sulfur compound as a cross-linking agent at extremely high temperatures of 140–200 °C.^11,12^ In sulfur-vulcanized rubbers, the network integrity is preserved not only by strong C–S and S–S bonds but also by exchangeable polysulfide bonds under mechanical loading or heating, enhancing the material’s extensibility and promoting strain-induced crystallization. This procedure can mostly provide access to improved organic solvent resistance, thermal resistance, creep resistance, and mechanical properties, yet it is challenging to obtain cross-linked PI with self-healing, recyclability, and other advanced functionalities due to the formation of an irreversible cross-linking network that restricts reconfiguration or repair of the network structure under mild conditions.

The trend strategy in the development of multifunctional cross-linked PI is to employ dynamic reversible cross-linking via transient bond formation, such as hydrogen bonds,^14–20^ ionic interactions,^9,21–29^ metal–ligand coordination,^30^ or molecular interdiffusion.^31^ These approaches have been proven feasible and can function as a cross-linking point that have association or dissociation spontaneous behavior under ambient conditions or certain environmental conditions to realize the damage-repair process that can significantly restore the original performance. In addition, by avoiding covalent cross-linking reactions that bridge the PI main chains, the recyclability feature can be achieved. Reversible ionic interactions are strong noncovalent bonds and a popular strategy to create PI films with self-healing ability at room temperature, without needing external stimuli like heat, ultraviolet (UV), pH changes, or healing agents.^22,24–26,28^ Several studies have demonstrated successful strategies to introduce ionic interactions into natural rubber (NR) networks composed mainly of 97% *cis*-1,4-PI by involving zinc dimethacrylate (ZDMA) as ionic cross-links, generating self-healing supramolecular NR molecules with strikingly high mechanical performance.^22,28^ Further investigation revealed that the ionic cross-linking reaction has limited self-healing abilities; therefore, the amount of self-healing at the same location can only occur once or a few times at the expense of decreasing mechanical and other related performances. Ionic interactions are also highly dependent and can even be disrupted by introducing strong electrostatic interactions, including dissociation upon the addition of water, polar salts, or strong solvents.^21,23,27^ In addition, the involvement of metal ions and difficult modification methodologies may narrow their applicability to fewer fields.^23,32^

Meanwhile, hydrogen bonding ranks as the second strongest noncovalent interaction, providing an optimal balance of mechanical strength and excellent reversibility, while avoiding undesirable repulsive forces between electrons or ions in the system.^14–18^ To maximize the construction of self-healing polymers, many researchers have introduced multiple hydrogen bonding systems, allowing easy tuning of the hydrogen bond cross-link density through the type, number, and position of bonding units. The 2-ureido-4-pyrimidinone (UPy) moiety, with its ability to form quadruple hydrogen bonds, provides efficient reversible cross-linking while minimizing covalent cross-link density. Incorporating UPy groups into the side chains of elastomers enhances elasticity, mechanical toughness, and strength, all while maintaining self-healing capabilities.^19,20,34^ For instance, Ding et al. reported the preparation of self-healing linear polyisoprene supramolecular elastomer (LPSE) through 3 consecutive steps of anionic polymerization, coupling reaction, and substitution reaction by deliberately grafting hydroxyl, isocyanate, and UPy groups onto the ends of PI backbone covalently.^19^ A significant stress hardening phenomenon of LPSE occurred after a certain deformation with a maximum mechanical strength of 1 MPa and maximum strain of 5.5%, while the self-healing process monitored under a polarizing microscope showed that the separation between two films could be reconnected without leaving any traces. A further complicated procedure was proposed by Lan et al., synthesis of anionically polymerized random styrene–butadiene rubbers (r-SBRs) followed by cross-linking process to incorporate dynamic 2­(6-isocyanatohexylaminocarbonylamino)-6-methyl-4­[1H]-pyrimidinone (UPy-NCO) and permanent hexamethylene diisocyanate (HMDI) into 1,4-olefins of butadiene units resulting in mechanical strength of 2.2 MPa, strain of 519%, and high self-healing efficiency of 100% at 60 °C for 24 h.^20^ Differently, Cordier et al. proposed a two-step synthetic route to introduce three types of complementary hydrogen bonding groups, namely amidoethyl imidazolidone, di­(amido ethyl) urea, and diamido tetraethyl triurea into aliphatic dimer acids.^18^ By utilizing the carboxylic acid ends to attach primary amine, secondary amine, tertiary amine, and amide functional groups, random hyperbranched polymers with excessive hydrogen bond density were formed. The self-healing process at room temperature only required 1 min of contact, and the tensile strength could be restored to 5.5 MPa. Among various types of hydrogen bonds, the interaction between carboxylic acid and amine groups has the potential to form strong hydrogen bonding forces. Considering the wide range of hydrogen bonding sources, developing hydrogen-bonded modified elastomers offers a simple, scalable, and cost-effective approach to meeting increasingly strict regulations. An additional challenge arises from the inherent polarity mismatches, as the incorporation of polar self-healing components (e.g., hydrogen bonding motifs or ionic groups) into the nonpolar PI matrix often results in phase separation or poor interfacial compatibility, complicating the design of homogeneous networks. Current approaches often rely on synthesizing PI blocks copolymerized with self-healing polymers from corresponding monomers,^1,15,16,19,20^ rather than directly modifying commercial PI. Therefore, our group desires to develop a novel approach to incorporate reversible interactions into the PI backbone through a simple one-step reaction to obtain cross-linked PI with additional dual-functionality, including self-healing and recyclability.

Benefiting from the availability of double bonds in the PI backbone, which can facilitate structural modification through radical reactions or bridging to new functional pendants, our group proposes a succinct strategy to chemically functionalize PI with l-Cysteine (LC) by a free radical-mediated thiol–ene reaction (Scheme 1). This reaction proceeds with regioselectivity consistent with anti-Markovnikov addition, specifically attaching the thiol group to the less substituted carbon of the PI double bonds.^35^ LC is a natural amino acid labeled as an environmentally friendly source and biodegradable polar compound, featuring reactive thiol, carboxylic acid, and amine functional groups. In this case, the reaction between PI and LC can be efficiently performed in a single step using a mixed solvent system of toluene and *N*,*N*-dimethylacetamide (DMAc) to overcome the differences in material polarity. As illustrated in Scheme 1a, with the aid of dicumyl peroxide (DCP) as a radical source, the reactive thiol group of LC can covalently link to the PI double bonds, resulting in the formation of insoluble functionalized PI-LC-X films in their own nonorganic solvent (i.e., toluene). The reactive carboxylic acid and amine groups undoubtedly present noncovalent cross-linking reaction through dynamic hydrogen bonds (Scheme 1b), which enables the reversible hydrogen bond breaking and reforming mechanism and results in improved elasticity, self-healing capability at room temperature, and excellent recycling-reshaping ability by relying on the acid hydrolysis process. The functionalized PI strategy employed in this study has also been proven to be effective in enhancing mechanical strength as the LC content increases, which is obviously due to the stronger and denser hydrogen bond cross-linking network. Therefore, this approach successfully overcomes the challenges associated with recyclability and polarity mismatch, providing a sustainable pathway applicable to other diene-based rubbers.

**Scheme 1:**
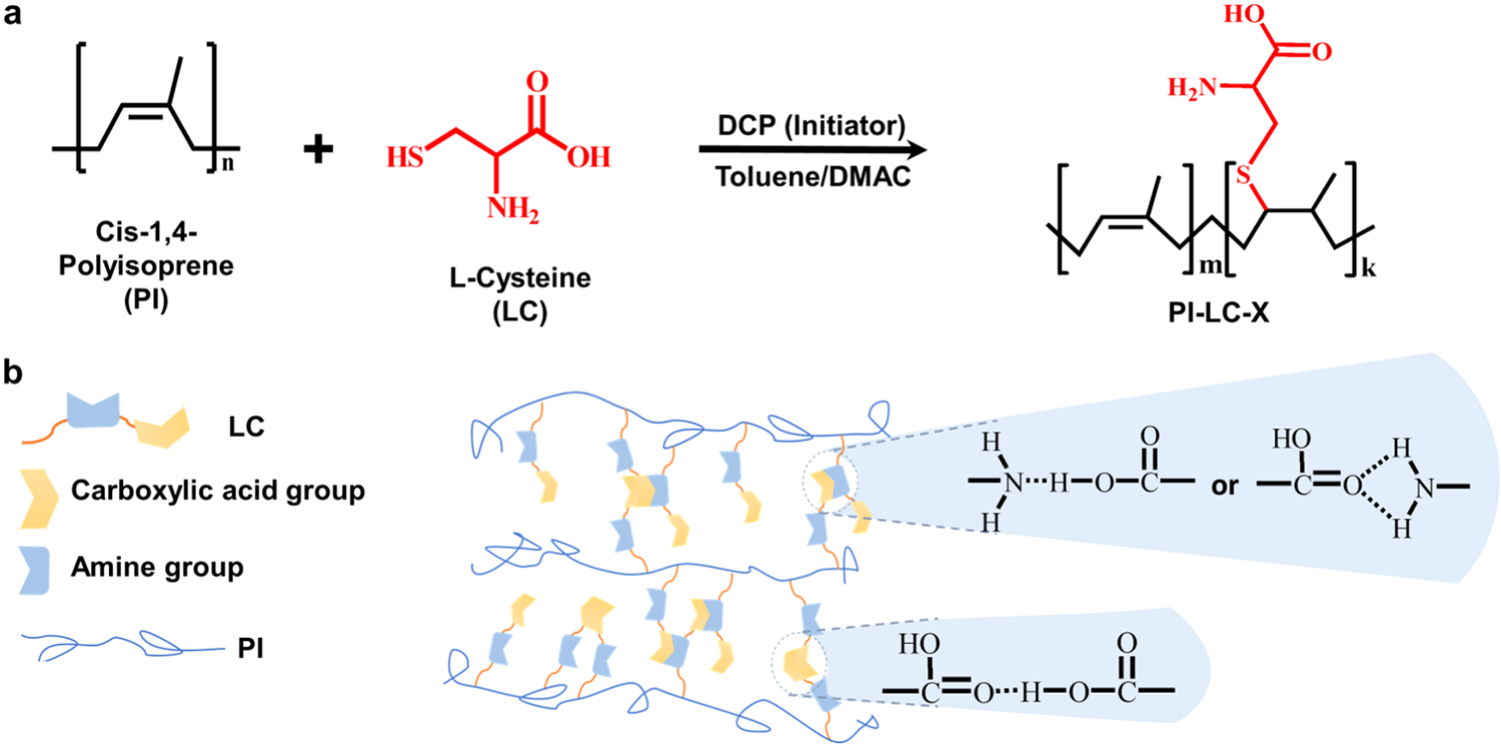
Conceptual Outline of This Study^a^

a (a) The reaction scheme of PI-LC-X and (b) a schematic representation of the dynamic cross-linking and self-healing mechanisms based on hydrogen bond interactions between the carboxylic acid and amine groups of LC.

## Results and Discussion

### Design and Characterization of PI-LC-X

A thiol–ene reaction proceeding via a free radical chain mechanism was employed to incorporate the LC compound into the PI double bonds using DCP as a thermal initiator.^35,36^ DCP undergoes homolytic cleavage upon heating to generate cumyloxy radicals. These radicals abstract hydrogen atoms from the thiol groups (R–SH) of LC, producing highly reactive thiyl radicals (RS^•^) and cumyl alcohol as a byproduct. The generated thiyl radicals preferentially add to the less substituted carbon (fewer alkyl groups) of the PI double bonds, leading to carbon-centered radical intermediates on the polymer backbone, which is consistent with the regioselective formation of the anti-Markovnikov product.^35^ The carbon radicals subsequently abstract hydrogen atoms from other LC thiols, regenerating thiyl radicals and effectively terminating chain growth at that addition site. This controlled radical propagation effectively prevents homopolymerization and unwanted chain extension and significantly suppresses cross-linking reactions between PI chains.

A preliminary assessment was conducted to validate the reaction effectiveness of the thiol group in LC linked to the PI double bonds. As a control, an experiment was performed under identical conditions without an initiator to ensure that no interaction occurs between PI and LC through simple mixing. As shown in Figure S1, both dried PI-LC-10 and control sample films were immersed in toluene. The PI-LC-10 film did not dissolve, confirming the formation of covalent thioether linkages between the LC and polymer chains. In contrast, the control sample dissolved completely, indicating the absence of a thiol–ene reaction between LC and PI. This solubility behavior confirms that the control sample retained properties identical to unmodified PI, verifying that effective functionalization in the PI-LC system requires initiator-driven thiol–ene chemistry rather than resulting from simple physical mixing.

The successful synthesis of the desired PI-LC-X was confirmed by ^1^H nuclear magnetic resonance spectrometry (^1^H NMR), Fourier transform infrared spectrometry (FTIR), and gel permeation chromatography (GPC) analyses. Figure 1a presents the ^1^H NMR spectra of PI, LC, and PI-LC-50 along with their chemical structures. PI-LC-50 was selected as the representative sample because it exhibited the most noticeable differences with and without LC, compared with PI-LC-10 and PI-LC-30. PI-LC-50 exhibits two new peaks at 1.30 and 3.04 ppm corresponding to the methylene proton of PI near the LC reaction site and methylene proton in thioether bond (CH**–**S**–**CH_2_), respectively, implying the SH group of LC effectively reacts with the double bond of PI forming covalent thioether linkages under the radical conditions generated by DCP. Note that certain proton signals corresponding to the LC structure are not observed in the ^1^H NMR spectra of PI-LC due to differences in solvent conditions. For detailed clarification, the comparative ^1^H NMR characterization results of PI-LC-X samples with varying LC ratios of 10, 30, and 50 are presented in Figure S2, revealing the appearance and increase or decrease in the cross-linked peak area. An interesting observation was that the intensity of the methylene proton signal associated with the thioether bond at 3.0 ppm decreased as the LC content increased. This attenuation is likely due to the high polarity of LC, which may cause poor solubility in the nonpolar deuterated chloroform (CDCl_3_-*d*) solvent, thereby reducing the visibility or resolution of the LC-related signals in the ^1^H NMR spectrum. Thus, quantification of LC bound to the PI double bonds was based on the integral area of the methylene proton peak at 1.30 ppm, which exhibits a significant increase in the integral area with increasing LC content (Figure S2), indicating more double bonds are obviously consumed by the thiol–ene reaction. The integral ratio of olefinic protons (C=CH, 5.16 ppm) to these methylene protons yields functionalization degrees of 10%, 30%, and 50% (mol % LC per PI repeat unit), matching our targets (Table 1).

**Figure 1:**
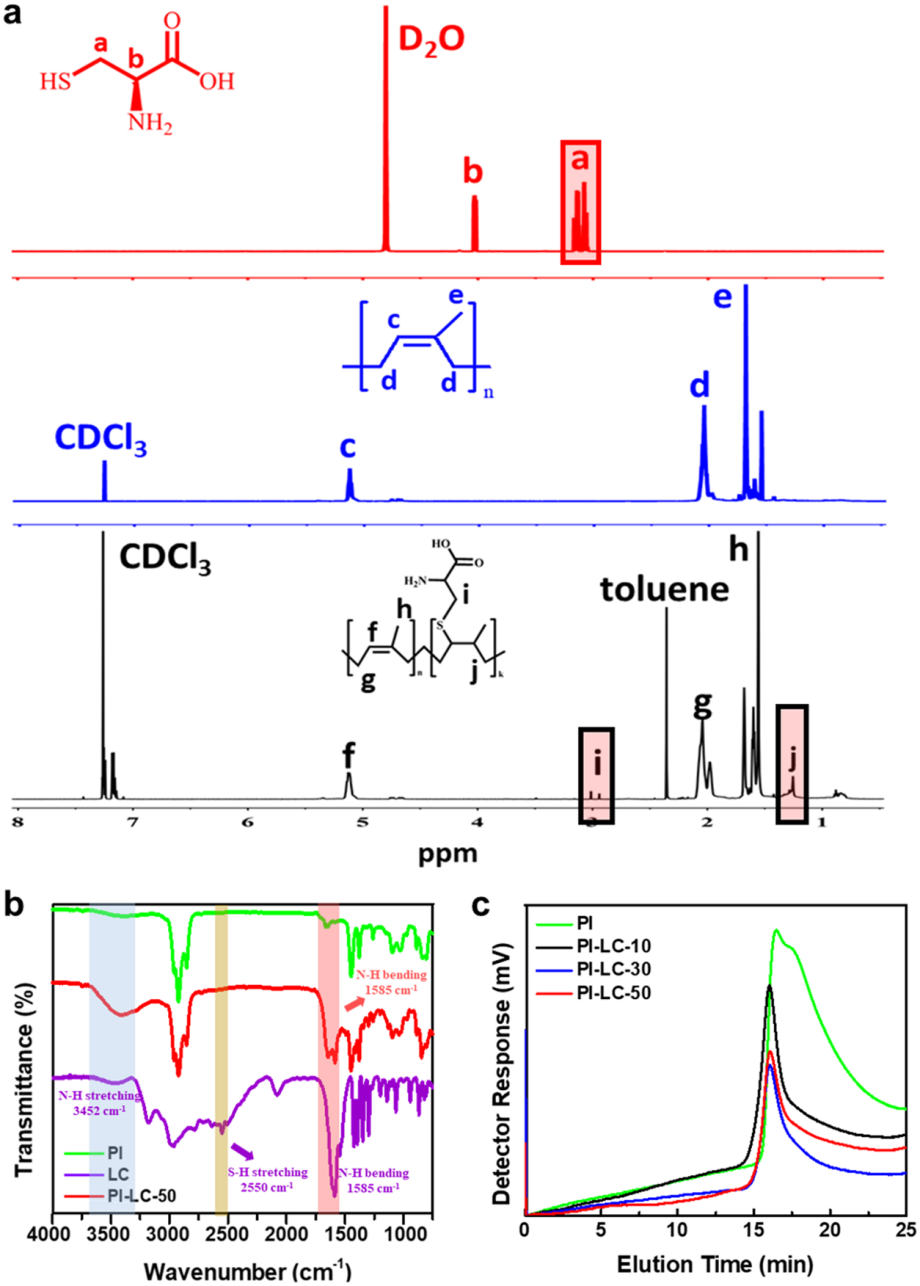
Characterizations via (a) ^1^H NMR spectra of LC in D_2_O (red line), PI in CDCl_3_-*d* (blue line) and PI-LC-50 in CDCl_3_-*d* (black line); (b) comparison of FTIR spectra between PI, LC, and PI-LC-50; and (c) GPC measurement of PI and a series of functionalized PI with different concentrations of LC.

**Table 1:** Characteristics of PI and PI-LC-X Samples Determined by ^1^H NMR and GPC Measurements

	H NMR analysis			GPC measurement data		
sample	Integral area ratio (C = C **H**: – C **H_2_**−)	functionalization degree (PI:PI-LC-X)	percentage of LC content attacking the PI double bond (%)	*M*_n_ (g mol^–1^)	*M*_w_ (g mol^–1^)	*Đ*
PI			-	23,300	26,500	1.14
PI-LC-10	1:0.23	0.90:0.10	10	32,200	33,600	1.04
PI-LC-30	1:0.85	0.70:0.30	30	34,000	35,000	1.03
PI-LC-50	1:1.97	0.50:0.50	50	36,200	37,500	1.04

Considering that not all LC proton signals are clearly observable in the ^1^H NMR spectra recorded in CDCl_3_, we further verified the successful synthesis of PI-LC-X by comparing the functional group profiles of PI, LC, and PI-LC-50 using FTIR spectroscopy. As displayed in Figure 1b, pristine PI exhibits characteristic absorption bands, including C=C stretching vibration at 1642 cm^–1^, C–H stretching vibration of methylene at 2855 and 2925 cm^–1^, and C–H stretching vibration of methyl at 2960 cm^–1^.^14,24^ Three major peaks were assigned to pristine LC including bending and stretching vibration of NH at 1590 cm^–1^ and 3452 cm^–1^, and a weak peak of S–H stretching at 2550 cm^–1^. Meanwhile, the PI-LC-X polymer retains most of the characteristic bands associated with the functional groups of both PI and LC. The absence of the characteristic S–H stretching peak at 2550 cm^–1^ in the PI-LC-X spectrum provides compelling evidence that the thiol groups of LC have been efficiently consumed through the covalent thiol–ene reaction with double bond PI. The disappearance of the -SH signal also demonstrates the effective incorporation of LC into the PI network, underscoring the ease and high efficiency of the synthetic approach. This conclusion is also verified by the increasing −NH peak with increasing LC content, as shown in Figure S3. These findings are consistent with Raman analysis, where the thiol (−SH) and alkene (C=C) bands appear at approximately 2551 and 1664 cm^–1^ in LC and PI, respectively (Figure S4). In PI-LC-X samples, the disappearance of the – SH band and the reduction of the C=C signal as the LC content increases confirm the consumption of reactive groups, establishing efficient thiol–ene network formation. Additionally, the relative decrease in the C=C band intensity quantitatively correlates with LC incorporation (Table S1). For instance, the residual C=C content in PI-LC-10 is 90.25%, indicating that approximately 9.75% of the double bonds were consumed, closely matching the 10% LC feed ratio. This proportional relationship between C=C consumption and LC addition demonstrates near-stoichiometric thiol–ene coupling and confirms efficient and controlled functionalization of the PI backbone. This strong agreement further validates the PI:LC composition determined by ^1^H NMR analysis and confirms the high efficiency of the thiol–ene functionalization reaction.

The number-average molecular weights (*M*_n_) and polydispersity index (*Đ*) values of PI and the varied ratio of PI-LC-X are shown in the GPC profiles of Figure 1c and are summarized in Table 1, respectively. Compared with pristine PI, the functionalized PI-LC-X exhibited a shift toward higher molecular weight regions and narrower peak shapes as the LC content increased, indicating a slight increase in molecular weight consistent with the successful covalent attachment of LC units to the PI backbone. The presence of a single well-defined GPC peak without overlapping or adjacent peaks indicates that the reaction predominantly results in side-chain functionalization rather than interchain covalent cross-linking or polymer branching. Additionally, their *Đ* values remain within a similar range to pristine PI, indicating a well-controlled functionalization process without significant broadening of the molecular weight distribution.

### Thermal Analysis and Characteristics of Cross-Linkable Dynamic Hydrogen Bonds

Thermogravimetric analysis (TGA) was conducted to elucidate the effect of LC on the thermal stability of PI (Figure 2a). In simple terms, the influence of increasing LC content on PI is evident from the weight loss observed at 230 °C. Both PI and PI-LC-10 show minimal weight loss of about 1.5 wt %, indicating that incorporating 10% LC onto the PI backbone does not significantly affect its thermal properties. In contrast, higher LC contents result in substantial weight loss of 22 wt % in PI-LC-30 and PI-LC-50, suggesting that the thermal behavior in this range is dominated by the LC component rather than the PI. Overall, the thermal decomposition profile is dependent on the composition of the materials incorporated into the PI. For more details, the first derivative thermogravimetric (DTG) analysis was conducted to facilitate the differentiation of elements decomposed in the host compound at each weight loss stage (Figures 2a and S5a–e). Both LC and PI exhibited a single main decomposition at 228 and 370 °C, respectively, corresponding to the primary degradation of their main chains (Figure S5a,b). It is anticipated that LC undergoes decomposition at a lower temperature than PI because of the instability of its sulfur-containing thiol group and the specific nature of its degradation process. In contrast, PI demonstrates superior thermal stability due to the strong carbon–carbon bonds present in its polymer backbone. The TGA and DTG traces for functionalized PI-LC-X (Figure S5c–e) exhibit two-stage decomposition, with initial degradation occurring at the temperature range of 229–236 °C (attributed to the breakage of the C–S bond of LC as the side chain of functionalized PI-LC-X) followed by the decomposition of PI backbone at 370–374 °C.

**Figure 2:**
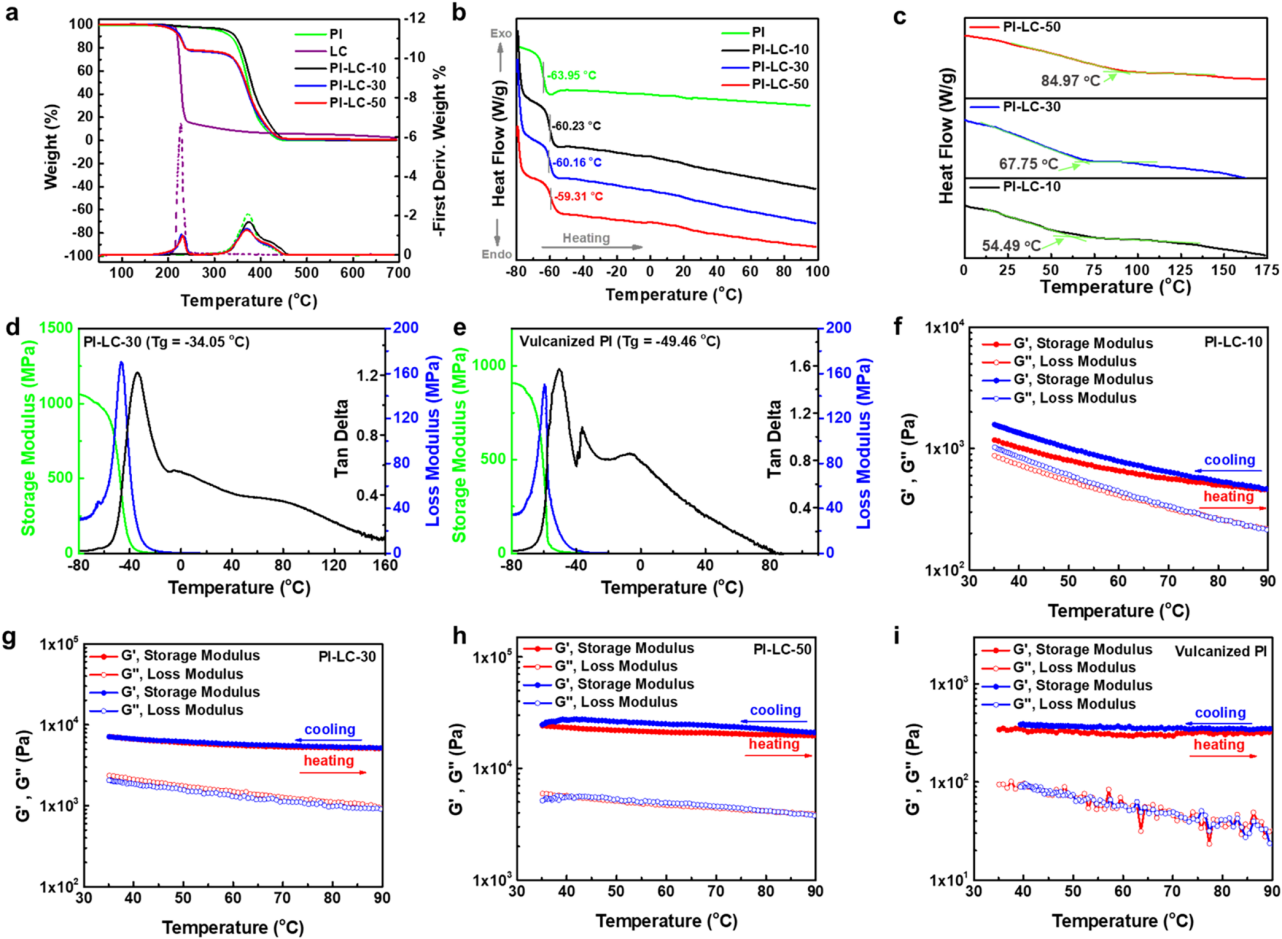
(a) TGA (solid lines) and corresponding DTG (dashed lines) curves of PI, LC, PI-LC-10, PI-LC-30, and PI-LC-50. DSC traces from the second cycle of each sample at a heating rate of 10 °C min^–1^ with operating temperature settings in the range of (b) −80 to 100 °C to determine the *T*_g_ values and (c) −75 to 175 °C to study the hydrogen bond breaking temperatures. Pre- and post-transition baselines appear in the −75 to 175 °C traces, with tangent intersections defining the respective transition temperatures. DMA curves of (d) PI-LC-30 and (e) covalently cross-linked PI via the vulcanization process. Temperature sweeps rheological properties with 1% strain amplitude, and ω = 1 rad s^–1^ of (f) PI-LC-10, (g) PI-LC-30, (h) PI-LC-50, and (i) vulcanized PI.

Since the self-healing capability of the PI-LC-X network critically depends on high polymer chain mobility as reflected by a low glass transition temperature (*T*_g_), to facilitate self-diffusing reversible noncovalent interactions, differential scanning calorimetry (DSC) was conducted to thoroughly characterize these thermal and dynamic properties. The *T*_g_ values of functionalized PI-LC-10, PI-LC-30, and PI-LC-50, as determined by DSC analysis, were found to be −60.23 °C, −60.16 °C, and −59.31 °C, respectively, which are similar to that of pristine PI at −63.95 °C (Figure 2b). These results indicate that increasing the LC content does not significantly affect the *T*_g_ values of the PI-LC-X systems. This behavior is attributed to the fact that the main chain structure remains predominantly PI, which is inherently characterized by a low glass transition temperature.^14,37^ Similar phenomena have been reported in supramolecular rubber systems featuring reversible hydrogen-bonded cross-links, where modifications to the side chain do not substantially alter the *Tg* value of the polymer backbone.^38^

Given that the previous DSC data shown in Figure 2b revealed only a slight increase in *T*_g_ values with increasing levels of cross-linked LC in the PI backbone, the temperature range was extended to 175 °C for further analysis. The contribution of hydrogen bonding to the thermal characteristics of PI-LC-X film is evident in Figures 2c and S6a–c. PI-LC-10 displays an endothermic transition at 54.49 °C attributable to hydrogen bond dissociation, with the endothermic heat flow increase reflecting the thermal energy required for this process. The thermal transition temperature increases with LC content, indicating the formation of progressively stronger and denser hydrogen-bonding networks that require a higher thermal energy to disrupt. Although these thermal features are subtle, particularly for PI-LC-50 that exhibits a transition temperature at 84.97 °C, their assignment to hydrogen-bond dissociation is supported by several observations. No corresponding glass transition or melting event appears in the 54–85 °C range, excluding conventional bulk thermal transitions. The characteristic temperature scales systematically with the hydrogen-bond content, while the small enthalpy change and broad endothermic signal indicate gradual hydrogen bond disruption. These subtle endothermic events occur within the typical hydrogen-bond dissociation range and are consistent with reports on stretchable semiconducting polymers, in which hydrogen-bond networks gradually weaken and depart at near 70–80 °C.^39^

Investigating the effects of PI functionalization (Figure 2d) versus vulcanization (Figure 2e) on thermal transitions using dynamic mechanical analysis (DMA) is crucial, as these two modification methods fundamentally alter the structure and properties of the polymer in distinct ways. For this study, the PI-LC-30 film was selected due to its superior mechanical strength and self-healing performance (as discussed in the following section, Figures 3a and 4). The preparation of PI-LC-X polymer film began with a drying process conducted in a hood for 24 h, followed by complete solvent elimination through heating at 70 °C under vacuum conditions for 6 h. This solvent removal process reduced the spacing between polymer chains, significantly enhancing the efficiency of hydrogen bond formation. Therefore, the proposed PI-LC-X film was formed purely without the addition of any cross-linking agents. The commercial PI in a viscous fluid form was vulcanized to produce a control sample for comparative evaluation. The vulcanized PI was prepared using 1,9-nonanedithiol (DT) as a cross-linking agent and reacted with pristine PI at 90 °C for 8 h. The amount of DT added is approximately 10% relative to the number of double bonds PI. During the vulcanization process, polymer chains formed a covalent network structure, transitioning PI from a viscous liquid to a solid film. For the vulcanized PI, both the storage modulus and loss modulus decrease sharply, and tanδ drops rapidly once the temperature exceeds *Tg*, reflecting the abrupt loss of mechanical integrity typical of irreversible covalent networks. In contrast, the PI-LC-30 film requires higher temperatures to disrupt the hydrogen bonds, so the side-chain hydrogen bonding continues to provide intermolecular interactions above *Tg*, resulting in a more gradual decrease in tanδ. This behavior highlights the dynamic and reversible nature of hydrogen bond cross-linking compared to the permanent covalent cross-links.^40,41^

**Figure 3:**
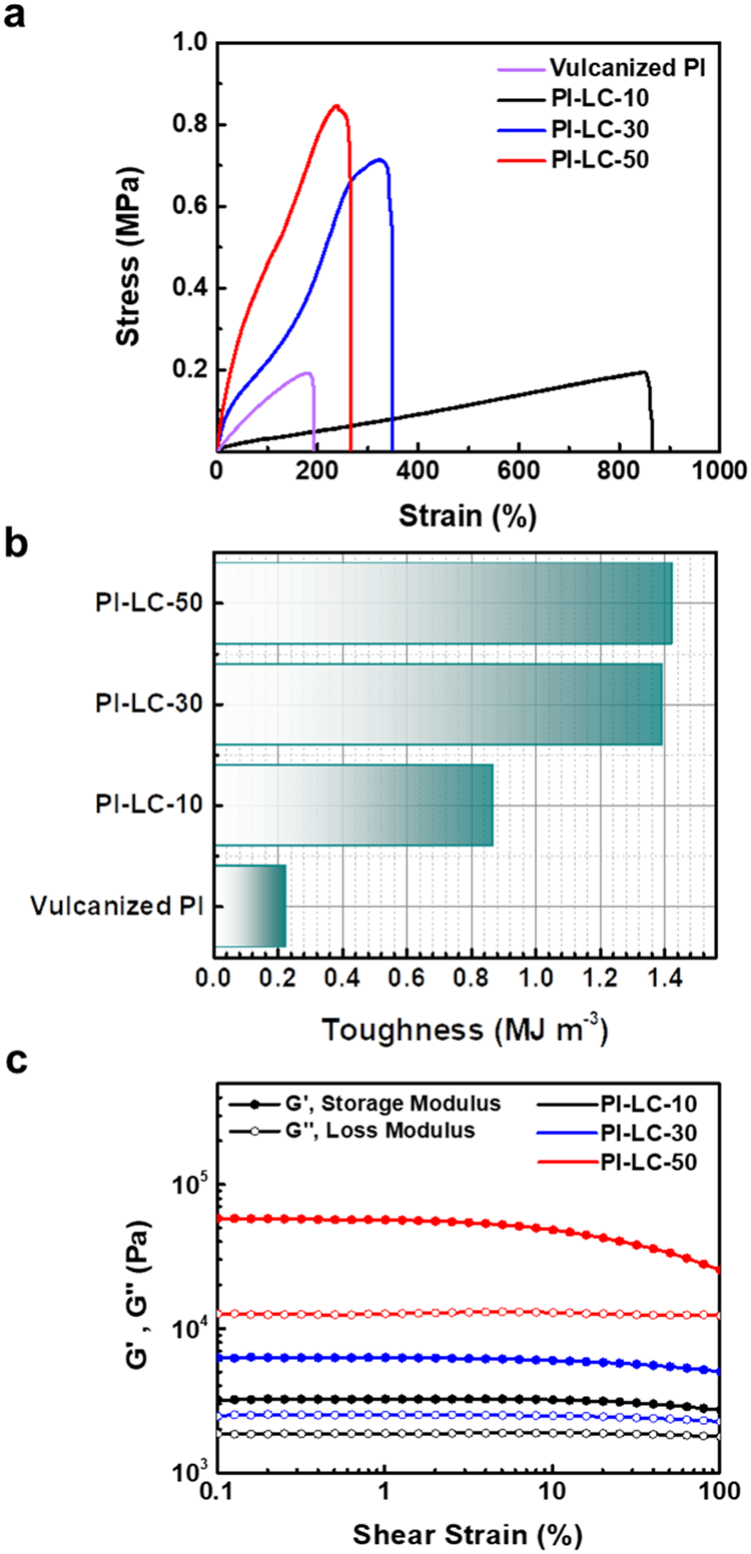
(a) Stress–strain curves of vulcanized PI and functionalized PI-LC-X and (b) their corresponding tensile toughness values. (c) Rheological amplitude sweep at frequency ω = 1 rad s^–1^ of the functionalized PI-LC-X. Note that the vulcanization procedure involves the use of a 10% concentration of additional cross-linker.

**Figure 4:**
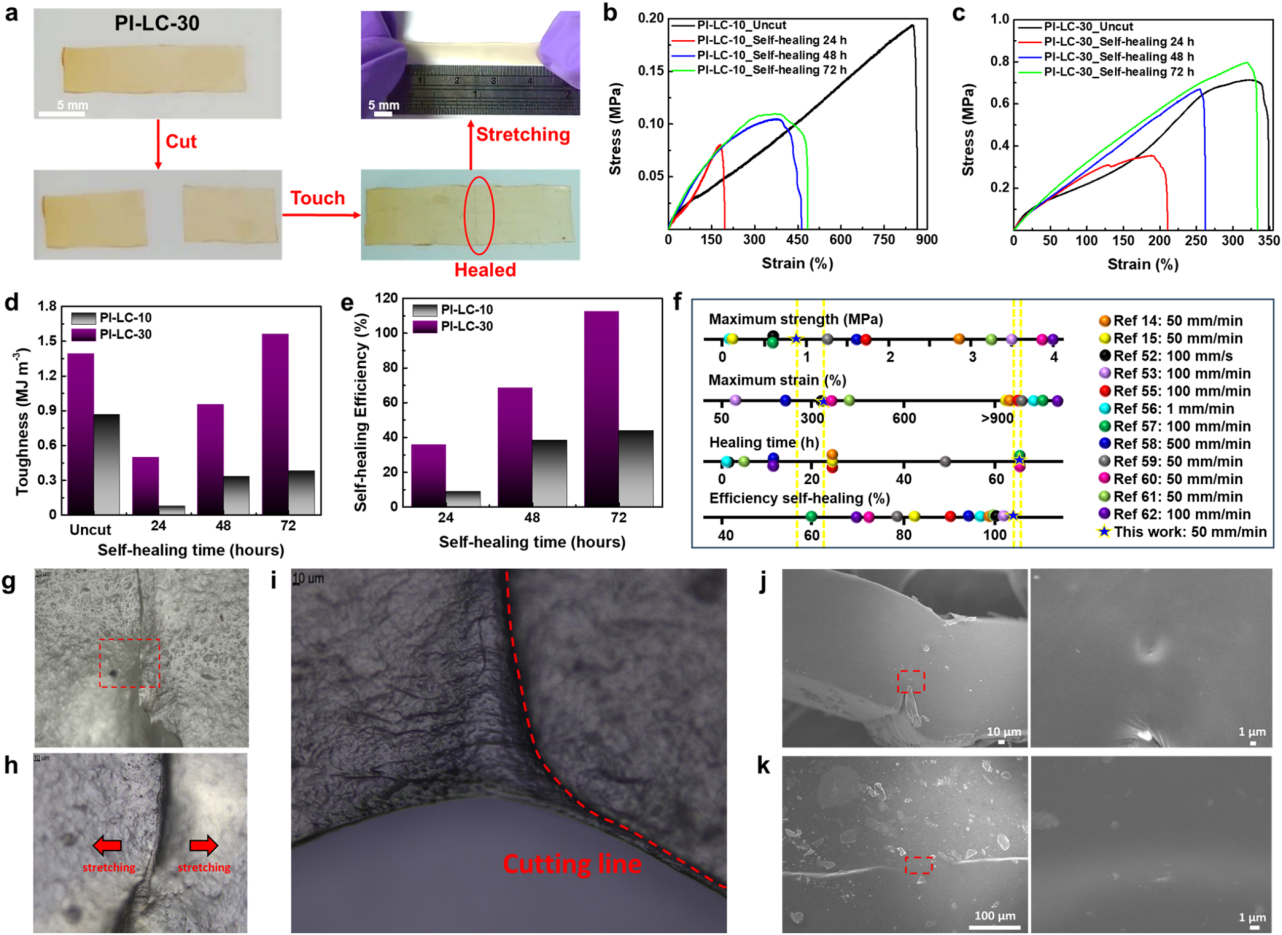
(a) Sequential photographs showing the self-healing process of PI-LC-30 film: initial cutting into two parts, recontacting of the separated surfaces, and healing after 48 h, with healing marks still slightly visible. (b) Stress–strain curves of PI-LC-10 film and (c) PI-LC-30 film after cutting and recontacting at room temperature for various durations. Bar charts summarizing (d) the toughness values and (e) the self-healing efficiencies of PI-LC-10 and PI-LC-30, measured from the uncut state and after 24, 48, and 72 h of self-healing. (f) Comparative analysis of PI-LC-30 and previously reported elastic and self-healing modified-PI and other types of elastomers in terms of mechanical performance after self-healing and corresponding self-healing efficiencies. The observation of self-healing PI-LC-30 thin film surface by OM after: (g) complete cutting and 48 h of contact, (h) self-healing PI-LC-30 film under slight stretching, and (i) stretching until cracks appear. SEM images of self-healed PI-LC-30 film: (j) cross-section and (k) surface morphology.

To further distinguish the effects of noncovalent cross-linking in functionalized PI compared to covalent cross-linking in vulcanized PI, a rheological analysis was conducted using temperature-sweep tests. During heating, the storage modulus (G′, elasticity) of PI-LC-10 exhibited a pronounced decrease, dropping from 1300 to 450 Pa (Figure 2f), which is attributed to the thermal dissociation of hydrogen bonds. Upon cooling back to room temperature, the storage modulus recovered and even slightly exceeded its initial value, indicating the reversible reformation of hydrogen bonds. With increasing LC content (Figure 2g,2h), the strengthened intermolecular interactions lead to only a slight decrease in storage modulus at elevated temperatures, exhibiting rheological behavior comparable to that of covalently cross-linked vulcanized PI samples (Figure 2i). This observation confirms that a higher LC content enhances the thermal stability of the hydrogen-bonded network, effectively replicating the characteristics of permanent cross-linking. More specifically, the rheological findings are consistent with the DSC data (Figure 2c), which show that samples with elevated hydrogen bond dissociation temperatures possess improved thermal stability, as indicated by their diminished sensitivity of storage modulus to temperature variations. These results imply that the polymer chains preserve strong hydrogen bonding interactions, even under high-temperature conditions.

Notably, temperature-sweep rheological cycling (Figure 2f–2i) reveals distinct hysteresis behavior among the samples. PI-LC-10, PI-LC-50, and the vulcanized PI display pronounced hysteresis, yet negligible hysteresis in PI-LC-30. This behavior reflects the dynamic balance of reversible interactions within the network. At intermediate LC content, PI-LC-30 achieves a balanced hydrogen-bond cross-link density, allowing relatively rapid dissociation and reformation of transient interactions in response to temperature changes. In contrast, sparse hydrogen bonds in PI-LC-10 yield insufficient dynamic reinforcement, while an excessive/dense hydrogen bonding network in PI-LC-50 prolongs relaxation via enhanced connectivity and bond persistence, thereby slowing network reorganization and leading to hysteresis. Likewise, the permanent covalent cross-links in vulcanized PI restrict chain mobility, further delaying relaxation dynamics and contributing to hysteresis. These results indicate that PI-LC-30 exemplifies a dynamically equilibrated network capable of a reversible response to thermal stimuli, underscoring the pivotal role of intermediate LC content in harmonizing mechanical robustness and self-healing capability with thermoreversibility.

### The Mechanical Performance and Viscoelastic Study of the Functionalized PI Film

The effect of an additional amount of LC on the mechanical behavior of PI was investigated using tensile testing. The tensile stress–strain curves of the vulcanized PI and PI-LC-10, −30, and −50 are compared in Figure 3a. Vulcanized PI and all PI-LC-X samples predominantly exhibit elastic deformation, indicating a reversible shape recovery. Sulfur-based PI cross-linking forms rigid covalent C–S linkages that bridge the carbon double bonds within the PI chains, yielding a vulcanized PI with a tensile strength of 0.19 MPa and a maximum strain limited to only 193%. While the mechanical strength of vulcanized PI is almost comparable to that of PI-LC-10, the latter demonstrates superior stretching capability of 865% due to the dynamic and flexible hydrogen-bonding networks, which can resist fracture under external forces and allow reversible reorganization under stress. This comparison of mechanical results underscores the critical role of cross-link type, material selection as a cross-linker, and structural architecture in determining the mechanical properties of PI. Precisely, PI-LC-10 demonstrates exceptional ductility and is capable of stretching up to 865% with a relatively low maximum stress of 0.2 MPa. As the proportion of LC linked to the PI double bond increases, the tensile strength rises to approximately four times that of PI-LC-10, while the elongation at break correspondingly decreases. This strength-ductility trade-off arises because a more densely constrained network restricts molecular slippage and segmental rearrangement during deformation, a phenomenon well-documented in systems where enhanced stiffness and strength are achieved at the expense of extensibility.^14–16,37,42,43^ For clarity and convenient comparison, the key mechanical parameters of the vulcanized PI and PI-LC-X samples are summarized in Table S2.

Interestingly, the tensile toughness values calculated from the total area under the stress–strain curve, which means encompassing both elastic and plastic deformation regions, showed an increase from PI-LC-10 to PI-LC-50 (Figure 3b). This trend indicates that PI-LC-50 progressively absorbs more energy during deformation, reflecting enhanced toughness and greater resistance to fracture under constant or multiple cyclic tests at a certain maximum strain.^44,45^ The observed trend in PI-LC-50 compared to other PI-LC-X, identified by decreasing mechanical strain alongside increasing mechanical strength and toughness, demonstrates the success in overcoming the traditional conflict of strength versus toughness and achieving acceptable damage tolerance levels essential for structural applications.^44^ In some cases, materials with lower strength and higher toughness, such as the stress–strain curve pattern displayed by PI-LC-10, are preferred in such safety-critical applications because they are less likely to fail prematurely under unexpected loads or stresses.^44,45^

To investigate the effect of LC content on the viscoelastic properties of functionalized PI-LC-X films, rheological analysis was performed by plotting shear strain against the storage modulus (G′) and loss modulus (G″). As shown in Figure 3c, the systematic increase in the storage modulus (G′) from ∼3 kPa (PI-LC-10) to ∼58 kPa (PI-LC-50) arises from progressive densification of the hydrogen-bonding network, where each LC unit contributes two donor/acceptor sites (−NH_2_/–COOH). Loss modulus (G″) increases proportionally but remains below G′ across all samples, confirming predominantly elastic behavior with sacrificial energy dissipation capacity. The inherent polarity mismatch between the nonpolar PI backbone and the polar LC side chains may promote localized aggregation, particularly at higher functionalization levels. Such interactions can lead to phase heterogeneity arising from hydrogen-bond-driven clustering of LC moieties. These clusters act as additional physical anchoring domains, further restricting chain mobility and enhancing the elastic response without inducing macroscopic phase separation. DSC analysis provides further evidence for the evolution of the hydrogen-bonding network. While the Tg values from the lowest (PI-LC-10) to the highest LC content (PI-LC-50) remain close to that of pristine PI (−63.95 °C; Figure 2b), the LC content-dependent endothermic transition associated with hydrogen-bond dissociation shifts to higher temperatures with increasing LC loading (Figure 2c). This shift indicates the formation of a denser and thermodynamically more stable hydrogen-bond network at higher functionalization levels. Closer examination reveals that PI-LC-50 exhibits an additional thermal transition near ∼20 °C (Figure 2b), resembling the thermal event observed for the pure LC compound at 22.20 °C (Figure S7). This low-temperature transition aligns with studies attributing it to zwitterion reorientation, thiol SH···S hydrogen bonding dominance, and backbone NH_3_^+^···O=C···HO hydrogen bond network distortions.^46,47^ As a highly ordered zwitterionic crystalline material, LC exhibits neither a Tg nor a melting point, instead undergoing direct endothermic decomposition upon heating.^48^ Accordingly, the observed thermal feature is more reasonably attributed to localized LC clustering or hydrogen-bonded microphase separation rather than to a conventional glass transition. This conclusion is corroborated by optical microscopy (10 μm scale) of PI-LC-X thin films, which reveals increasing surface roughness and the presence of irregular LC aggregates that become more numerous with higher LC content (Figure S8). Together, these structural features explain the observed rheological trends; as LC content increases, G′ rises due to the greater density of hydrogen-bond-mediated physical cross-links, which restricts chain mobility and reinforces the elastic network. Simultaneously, G″ increases as these dynamic hydrogen bonds undergo reversible breaking and reformation, introducing additional relaxation pathways and energy dissipation. Overall, the viscoelastic behavior of PI-LC-X films reflects the combined effect of progressive hydrogen-bond cross-linking and localized LC-rich aggregation, explaining the critical role of LC functionalization in tuning the viscoelasticity and mechanical stiffness.

### Self-Healing Evaluation through Mechanical Testing and Surface Morphology Analysis

Imparting the self-healing capability to PI is crucial for enhancing the durability, safety, and environmental sustainability of materials across various applications. In the functionalized PI-LC-X network, the main self-healing mechanism relies on reversible noncovalent interactions originating from the reactive −NH_2_ and −COOH groups of LC (Scheme 1b). The low *T*_g_ of the PI-LC-X network indicates a high molecular mobility at ambient conditions (Figure 2b), thereby promoting autonomous self-healing without the need for external stimuli. Figure 4a illustrates the self-healing process of the PI-LC-30 film within 48 h to demonstrate the hydrogen-bond-mediated self-healing behavior. A rectangle PI-LC-30 film with the dimensions of 0.5 mm in thickness, 5 mm in width, and 20 mm in length was cut in the middle using a razor blade and then reconnected with slight pressure for 5 s on both sides of the fractured surface. Notably, the damaged sample spontaneously self-heals at room temperature with visible scars still remaining and can be stretched to more than twice its original length after recovery. The quantitative self-healing efficiency of PI-LC-10 and PI-LC-30 was assessed by comparing the toughness values of the healed films with those of the uncut films, thereby accounting for both stress and strain recovery. As shown in Figure 4b,4c, the mechanical properties of the healed samples improved significantly as the healing time extended to 72 h, where the self-healing efficiencies of PI-LC-10 and PI-LC-30 were 44% and over 100%, respectively. Among the tested compositions, PI-LC-30 exhibited the most excellent self-healing behavior with the recovering maximum tensile stress of 0.96 MPa and strain of 261%, indicating the healed film was even tougher. Detailed toughness values and self-healing efficiencies measured at various time intervals of 24, 48, and 72 h during the self-healing process are presented in Figure 4d,4e, respectively. Further analysis of the stress–strain curves for both uncut and healed functionalized PI-LC-10 and PI-LC-30 films (Figure 4b,4c) reveals an increase in mechanical strength after the polymers were allowed to self-heal at room temperature for 24, 48, and 72 h without any external intervention. This phenomenon is attributed to ongoing dynamic hydrogen bond reorganization in the functionalized PI-LC-X network, which autonomously facilitates the diffusion, interpenetration, and reformation of dynamic hydrogen bonds at room temperature postdamage, resulting in gradual restoration and even enhancement of mechanical integrity as the healing process progresses. Similar cases have been reported in other literature.^49–53^ For example, in polyurethane elastomers and polyurea networks, hierarchical hydrogen bonding and dynamic imine bonds enable efficient self-healing and strengthening at room temperature.^50,51^ As the material is left undisturbed, the reversible bonds continue to reform and optimize the network structure, resulting in increased tensile strength, toughness, and elongation at break over time. This process is further facilitated by a noncrystallized structure and sufficient chain mobility, which allow for effective interdiffusion and bond reformation at ambient temperature.^51–53^

Additionally, during the tensile test of self-healed PI-LC-X films, the tear resistance of the self-healed region was detected, demonstrated by its ability to withstand slight damage and continue stretching until complete rupture. This property contributes to the enhanced mechanical strength observed in the tensile test of the healed PI-LC-X film. To prove this, tear resistance tests were performed on PI-LC-10 and PI-LC-30 films by cutting one-third of the original film width and subsequently evaluating their mechanical properties. As shown in Figure S9a,b, the maximum tensile strain of PI-LC-10 and PI-LC-30 films reached approximately 300% and 200%, respectively. At equivalent strain levels, both notched films exhibited notable increases in the mechanical strength after testing. These results demonstrate that PI-LC-10 and PI-LC-30 films under tear conditions can bear substantial tensile loads and stretch to more than twice their original length without breaking, indicating effective tear resistance and a strengthened mechanical integrity.

Unlike other functionalized PI-LC variants, PI-LC-50 exhibited no self-healing capability. This arises from excessive hydrogen-bonding density driving LC-rich cluster formation (as evidenced by DSC in Figures 2b,2c and S7 and OM images in Figure S8). These clustered domains act as long-lived physical cross-links that slow the hydrogen bond network relaxation and limit free functional groups at fracture surfaces, hindering interfacial rebonding despite the backbone mobility above *T*_g_.^54^ This mechanism aligns with the pronounced DSC endothermic shifts to higher temperatures with increasing LC content (Figure 2c), as well as the rheological hysteresis observed in temperature-sweep cycling (Figure 2f–2i), where dense bonding in PI-LC-50 impedes network reorganization compared to the dynamically balanced PI-LC-30. As illustrated in Figure 4f, while the mechanical properties of our functionalized PI-LC-30 do not exceed those of all compared materials, they remain highly competitive among reported elastomers and exhibit excellent self-healing efficiency.^14,15,52,53,55–62^ Therefore, these properties still represent a favorable balance, especially considering that the overarching goal of this research is to develop sustainable functionalized PI with the addition of dual features consisting of self-healing and recyclability (the latter will be discussed in a later section), concurrently improving elasticity and mechanical performance.

Owing to its excellent self-healing performance, PI-LC-30 was selected as the representative sample for detailed evaluation of self-healing behavior using Optical microscopy (OM, Figure 4g–4i). After 48 h, the healed region was identified by the cut marks remaining on the film (Figure 4g). Although cracks at the fracture interface persisted, a slight stretching of the PI-LC-30 film indicated that the internal regions of the fractured surfaces had undergone autonomous healing (Figure 4h). Upon further stretching, new cracks formed and the fracture surface shifted adjacent to the original cutting line, confirming that the repaired interface retained good mechanical strength after self-healing (Figure 4i). Scanning electron microscopy (SEM) provided further insight into the self-healing interface of the PI-LC-30 film. As shown in Figure 4j,4k, the cracks between the two fractured surfaces are nearly indistinguishable, confirming the effectiveness of the self-healing process.

### The Mechanical Recovery Study Based on Reversible Sacrificial Hydrogen Bonds via Cyclic Tensile Testing of PI-LC-X Films

In addition to assessing mechanical and self-healing properties, cyclic tensile testing of both PI-LC-10 and PI-LC-30 films offers valuable insights into the underlying mechanisms of hydrogen bond dynamics. During mechanical deformation, these functionalized PI-LC-X films experience strain-induced dissociation and recombination of hydrogen bonds, a process that plays a critical role in determining their mechanical response. Specifically, reversible hydrogen bonds formed between carboxylic acid and amine groups serve as multiple sacrificial bonds, rupturing under applied stress to dissipate energy and reforming upon unloading, thereby facilitating structural recovery and restoration of mechanical properties.

Figure 5a–d compares the stress–strain curves from five consecutive cyclic tensile tests with a fixed maximum strain of 200% for PI-LC-10 and PI-LC-30 films, both in their original state and after 72 h of self-healing. All samples demonstrated remarkable recovery, characterized by small hysteresis loops and residual strains below 25%, which fully reverted to their original dimensions after brief relaxation periods of less than one minute. These results emphasize the dynamic nature of reversible hydrogen bonds that are capable of repeated breaking and reforming throughout successive loading–unloading cycles. The repeated cycling performance of the healed PI-LC-10 (Figure 5b) and PI-LC-30 (Figure 5d) even surpasses that of the original samples without self-healing treatment, as evidenced by the overlapping stress–strain curves of the first cycle, indicating the high effectiveness of dynamic hydrogen bonds in facilitating self-healing. The eventual stabilization of the mechanical properties of healed PI-LC-30 after multiple cycles suggests that the hydrogen bonding network reaches an equilibrium state, where dissociation and recombination processes occur at comparable rates. This equilibrium contributes to the robust elastic performance and durability of healed PI-LC-30.

**Figure 5:**
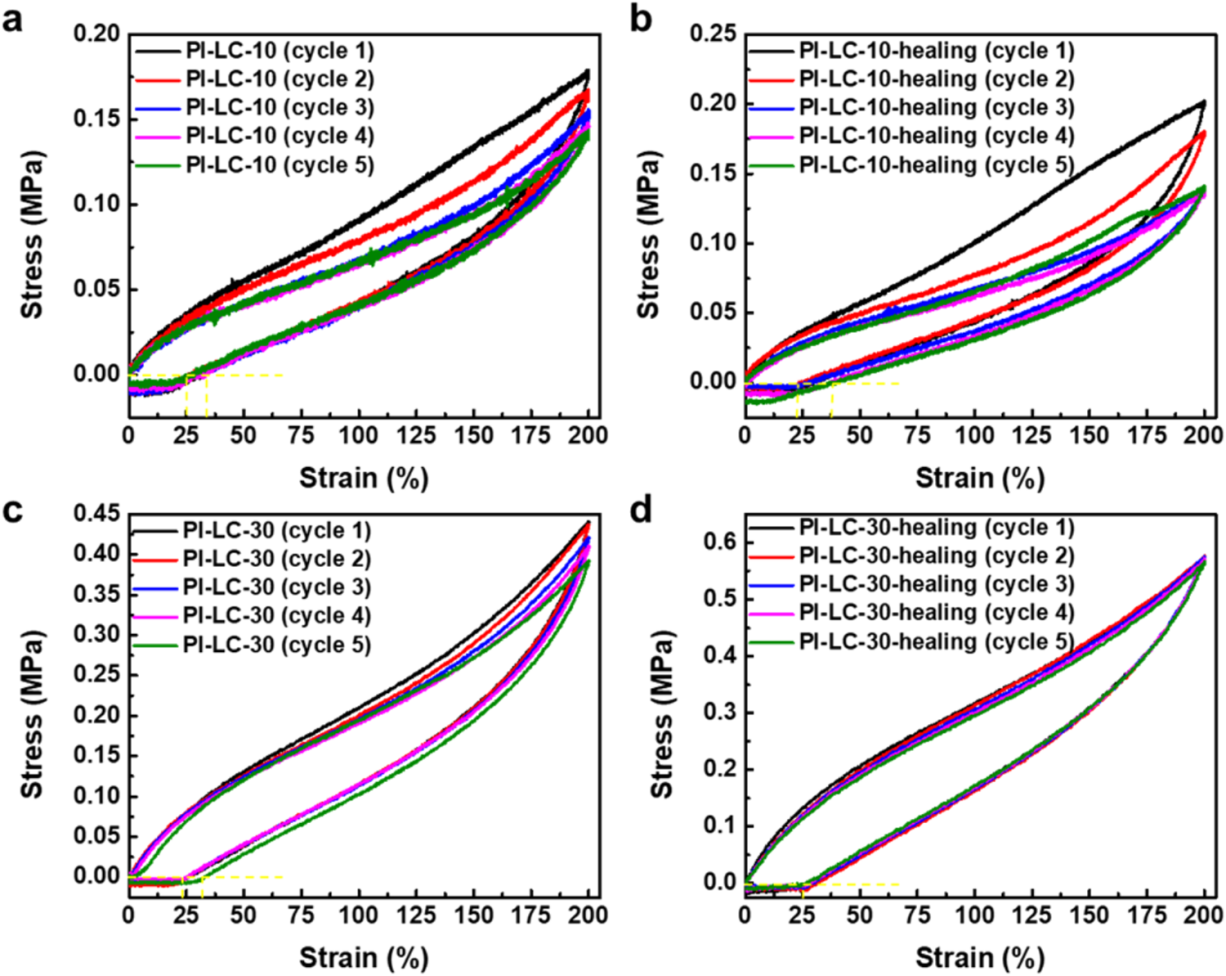
Cyclic stress–strain curves from five consecutive tests at 200% maximum strain with a 1 min rest between cycles for: (a) original PI-LC-10, (b) healed PI-LC-10, (c) original PI-LC-30, and (d) healed PI-LC-30.

### Recyclable Property

The inherent polarity of LC and its capability to form strong and dense hydrogen-bonding networks significantly increase the insolubility of functionalized PI-LC-X, posing significant challenges for material recycling. To overcome this, we implemented chemical hydrolysis under acidic conditions to efficiently disrupt the dense hydrogen-bonding network. Under acidic conditions, protons (H^+^) protonate electron-rich functional groups such as −NH_2_ and −COOH within LC, weakening intermolecular hydrogen bonds and thereby facilitating dissolution of the polymer network. Given the superior mechanical properties, self-healing ability, and recoverability, PI-LC-30 was selected as the representative system. Small pieces of PI-LC-30 film were dissolved in acidic tetrahydrofuran (THF) at 65 °C for 12 h. During this acidic treatment, the amine groups of LC are temporarily protonated to ammonium species, which disrupts the hydrogen-bonding network and facilitates dissolution. The resulting solution was then precipitated in methanol to remove residual acid, followed by two stages of solvent removal (drying at room temperature for 24 h and heating at 70 °C under a vacuum for 6 h) to obtain a regenerated PI-LC-30 film (Figure 6a). These acid elimination and solvent removal processes enable spontaneous deprotonation and restoration of the neutral amine functionality, leading to the reformation of dynamic hydrogen bonding networks and efficient recovery of the material without loss of structural integrity. The chemical integrity postrecycling was confirmed by ^1^H NMR after three cut-recycle cycles (Figure S10), showing unchanged thioether methylene signal (3.06 ppm) with no new peaks, broadening, or chemical shifts, indicating complete restoration of neutral amine functionality rather than persistent ammonium species. This reversible protonation/deprotonation process aligns with cysteine-based polymer systems reported by Tsuchiya et al., where dynamic interactions enable material reprocessing upon treatment.^63^ Additionally, the preserved methylene proton signals of the PI-bound LC (1.30 ppm) confirm covalent bond integrity during acid treatment. Consequently, recycled films require no refunctionalization, as the hydrophobic PI backbone effectively shields these linkages from hydrolytic attack. Mechanical tensile testing further demonstrates the robustness of recycled PI-LC-30 films (Figure 6b). The nearly identical stress–strain curves before and after three recycling cycles confirm the effective recovery of mechanical properties. Quantitative analysis (Table S3) reveals only minor deviations in maximum strain, tensile strength, and toughness. These slight differences arise from the random reformation of dynamic hydrogen-bonded cross-links during thermal film molding, which leads to slight variations in cross-link density and distribution compared with the original network. Overall, the recycled films with identical mechanical properties without refunctionalization confirm complete network recovery and the preservation of the original covalent structure. Such excellent recyclability emphasizes the durability and reusability of the PI-LC-X networks, demonstrating their strong potential as sustainable materials for future applications.

**Figure 6:**
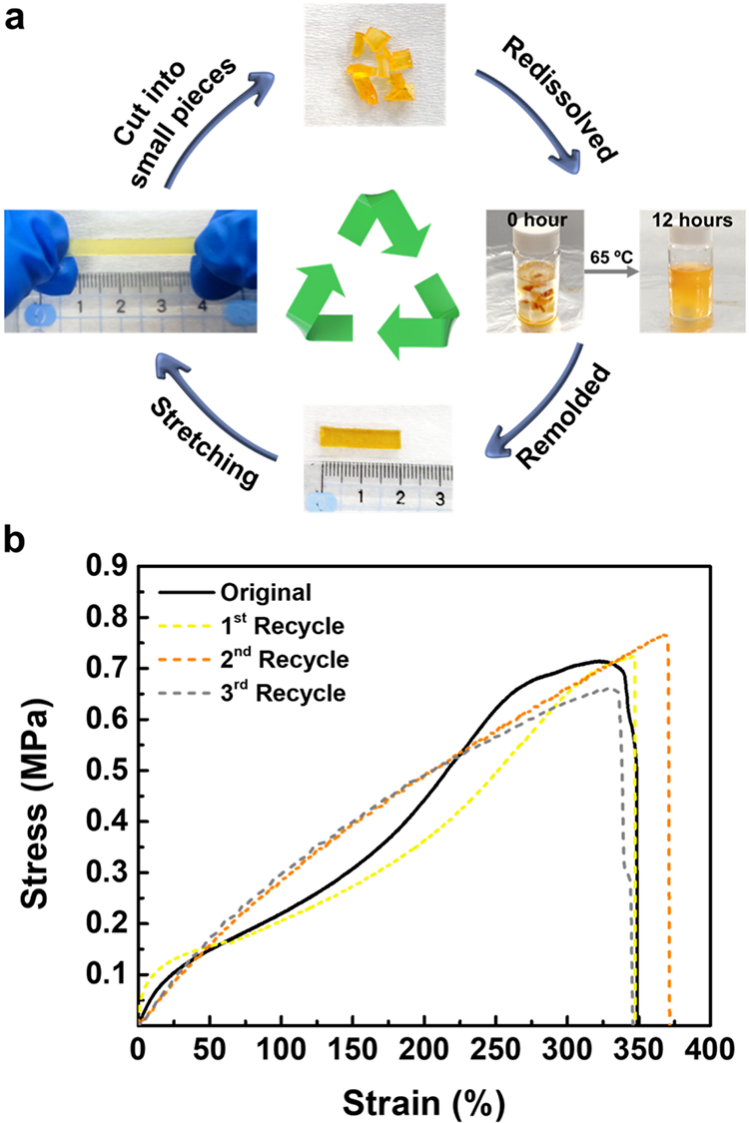
(a) Illustration of the recycling process and (b) stress–strain curves of recycled PI-LC-30.

## Conclusion

This study highlights the effective synergy between covalent and noncovalent interactions resulting from LC linked to the PI double bonds. Covalent thiol–ene linkages impart robust structural integrity, while dynamic noncovalent interactions serve as reversible sacrificial bonds, enhancing both mechanical strength (transforming PI from a low-viscosity fluid into a solid PI-LC-30 film with mechanical strength of 0.71 MPa, strain of 348%, and toughness of 1.39 MJ m^–3^, where these mechanical properties remain competitive with existing elastomers) and elasticity (as evidenced by small hysteresis loops and consistent overlap of stress–strain curves over five cycles). These synergistic interactions also confer advanced functionalities, including a high self-healing efficiency of over 100% at room temperature and excellent recyclability, as demonstrated by its ability to retain mechanical properties after repeated cutting, dissolving in acidic THF solution, neutralization, and molding. These results underscore the success of integrating LC into the PI via the thiol–ene reaction to create robust, elastic, self-healing, and recyclable polymer networks, thus establishing a promising pathway for developing next-generation sustainable rubber-like materials with enhanced durability and functionality. Furthermore, the use of readily available commercial chemicals and a straightforward preparation process make this method highly feasible for industrial-scale production.

## Experimental Section/Methods

### Materials and Chemicals

*Cis*-1,4-polyisoprene (PI, unsaturation 92 mol %, *M*_w_ = 35,000), l-cysteine (LC, 97%), and dicumyl peroxide (DCP, 98%) were supplied by Sigma-Aldrich. *N*,*N*-Dimethylacetamide (DMAc, 99%) and toluene (99.5%) were purchased from Alfa Aesar. Tetrahydrofuran (THF, 99%) was purchased from ECHO CHEMICAL CO., Ltd. Sulfuric acid (H_2_SO_4_, 95–98% Reagent grade) was purchased from Scharlab S.L. Deuterated chloroform (CDCl_3_-d) and deuterium oxide (D_2_O) were purchased from Sigma-Aldrich.

### Synthesis of PI-LC-X

Modification of PI-LC-X (where *X* means the ratio of the number of LC attached to the PI double structure, consisting of 10, 30, and 50) was synthesized through a radical-mediated thiol–ene reaction. A mixture solution of 1.120 g of PI, 0.0756 g of DCP, and 14 mL of toluene in a glass vessel was degassed with argon (Ar) for 20 min. On the other hand, a certain amount of LC (depending on the targeted content, 0.206 to 1.030 g) was dissolved by 14 mL of DMAc at room temperature under an Ar atmosphere in a double-neck flask connected to a condenser. The solution mixture of PI in the glass vessel was transferred to the double-neck flask containing solution LC via a purged syringe. The reaction was carried out in a preheated oil bath at 115 °C for 24 h. After cooling to room temperature, the solvent was removed under vacuum; the product was precipitated three times with methanol, redispersed in toluene, and stored in a tightly sealed glass bottle. Yellow viscous liquid of PI-LC-X polymers was obtained in >95% yield. Purity and composition were checked by ^1^H NMR spectroscopy.

### Preparation of PI-LC-X Thin Films

The PI-LC-X in toluene solution was poured into a Teflon mold and dried in a hood for 24 h. To completely remove the solvent, a further heating process was applied at a moderate temperature of 70 °C under vacuum conditions for 6 h, producing a PI-LC-X film. These two steps of the solvent removal process promote the formation of intermolecular hydrogen bonds.

### Characterizations Method

The structures, molecular weights, and polydispersity index (*Đ*) of the PI-LC-X were determined by ^1^H nuclear magnetic resonance spectrometer (^1^H NMR), Fourier transform infrared spectrometer (FTIR), and gel permeation chromatography (GPC). Solution-state ^1^H NMR spectra of the PI-LC-X were recorded on a Bruker AVIII HD-600 type 400 MHz spectrometer at 25 °C. Chemical shifts (δ) are reported in ppm relative to the residual protium solvent peak in CDCl_3_-*d* (δ 7.26). The FTIR spectra were collected with a Tracer-100 spectrometer (Shimadzu, Japan) at 25 °C with an accumulation of 64 scans. MicroRaman measurement (JASCO 5100 spectrometer) was conducted by scanning the *XY* plane with a laser excitation wavelength of 532 nm and carried out using a laser power of 4.0 mW. The molecular weights of PI-LC-X, including number-average molecular weight (*M*_n_), weight average molecular weight (*M*_w_), and Đ, were determined by GPC using tetrahydrofuran (THF) as eluent at a flow rate of 1 mL min^–1^ at 40 °C. A Waters 1515 HPLC pump equipped with a Waters 2414 RI refractive index detector was used with reference to a range of polystyrene standards. The thermal stability was measured by TA Instruments TGA550 type in a nitrogen atmosphere. The thermal transition behavior is analyzed by a TA Instruments Discovery DSC 25 type differential scanning calorimeter (DSC) at a heating rate of 10 °C min^–1^ under a nitrogen atmosphere to understand the effect of hydrogen bonds on the material. All of the mechanical tensile properties were conducted at a rate of 50 mm min^–1^ by a Shimadzu-EZ-EX instrument. An Anton Paar’s MCR 92 rheometer equipped with “Melt Linear Viscoelastic Range (LVER)” and “gelification” procedures were used to further measure the viscoelastic behavior of materials, including the measurements of storage modulus (*G*′) and loss modulus (*G*″) to verify the formation and breakage of hydrogen bonds.

### Evaluation of the Self-Healing Behavior

To determine the self-healing efficiency of the PI-LC-X film, rectangular specimens measuring 0.5 mm in thickness, 5 mm in width, and 20 mm in length were prepared. Each specimen was cut in the middle, and the two halves were then gently pressed together for 5 s to allow merging at the interface. The healing time varied between 24, 48, and 72 h under ambient conditions with an average relative humidity value of 55 ± 5% at 25 ± 2 °C, as measured by a large-screen LCD digital electronic temperature and humidity meter WD-5016. Tensile tests were performed on both healed and uncut PI-LC-X film specimens. Healing efficiencies were estimated from the ratio of the toughness of the healed samples to that of the uncut ones. The toughness value was calculated by integrating the area under each stress–strain curve using the trapezoidal rule. Optical microscopy (OM) and scanning electron microscopy (SEM) were used to observe the self-healing ability of the PI-LC-X thin film under microscopic conditions. In detail, the preliminary self-healing was observed at 10× and 20× magnifications of the film through BX53 M type OLYMPUS OM. SEM of the JEOL JSM-6330F type was used to observe the self-healing of the material in a vacuum environment with a resolution of 1.5 nm and magnifications of 300×, 1500×, 3000×, and 10000×.

### Rheological Measurements

The viscoelastic properties of the PI-LC-X films were characterized by using an Anton Paar MCR 92 rheometer equipped with parallel-plate geometry (8 mm diameter). To achieve reliable measurements and minimize sample slippage in these robust and elastic networks, uniform solid films (0.5 mm thickness) were carefully positioned and gently compressed to ensure a tight plate contact and sufficient interfacial friction for accurate shear stress transmission. Storage (*G*′) and loss (*G*″) moduli were determined through strain amplitude sweeps (0.1–100% strain) at a fixed frequency of ω = 1 rad s^–1^, which was confirmed to lie within the linear viscoelastic region (LVER) through strain sweep tests. Operating within the LVER minimizes the risk of structural disruption or interfacial slip caused by excessive deformation. Subsequently, measurements employed a fixed frequency of ω = 1 rad s^–1^ across 25–90 °C to probe temperature- and frequency-dependent hydrogen bonding dynamics and confirm thermal reversibility of the functionalized PI networks.

### Acid Hydrolysis of PI-LC-X

The procedure began by cutting 200 mg of PI-LC-30 into small pieces, which were then dissolved in a solvent mixture of 1 M aqueous H_2_SO_4_ and THF at a volume ratio of 1:2 at 65 °C for 8 h with stirring at 1000 rpm. Subsequently, the solvent was removed using a rotary vacuum evaporator to eliminate THF, followed by precipitation of PI-LC-X with methanol to remove aqueous H_2_SO_4_. The resulting PI-LC-X was placed into a Teflon mold and dried in a vacuum oven at 70 °C for 6 h. Thus, the procedure for preparing PI-LC-X film was repeated.

## Supplementary Material



## Notes

Photographs of PI-LC-10 and control samples; ^1^H NMR, FTIR, Raman, TGA, and DSC data for all synthesized PI-LC-X compounds; OM images; mechanical tensile testing data; and comparative performance tables (PDF)
