# Supplementary material for: Introduction of Self-Healing and Recyclable Properties into Functionalized Polyisoprene Rubber via Thiol–Ene Reaction
Source: ACS Polym Au. 2026 Mar 6;6(2):645–58. doi: 10.1021/acspolymersau.6c00009 (PMC13067166; doi:10.1021/acspolymersau.6c00009)
Supplement: Supplementary file 1 [file lg6c00009_si_001.pdf]

Supporting Information

Introduction of Self-Healing and Recyclable Properties into Functionalized Polyisoprene Rubber via Thiol-Ene Reaction

Yan-Sin Huang[†1], Livy Laysandra[†1], and Yu-Cheng Chiu[*1,2]

[1]Department of Chemical Engineering, National Taiwan University of Science and Technology, No.43, Sec. 4, Keelung Rd., Da'an Dist., Taipei City 10607, Taiwan.
[2]Advanced Research Center for Green Materials Science and Technology, National Taiwan University, Taipei 10617, Taiwan.

[†]Equally contributed to the work

*Corresponding author: ycchiu@mail.ntust.edu.tw

List of Contents for the Supporting Information

S1. Photographs demonstrating the PI-LC-10 and control sample soaked in toluene.............................2
S2. The characterization of functionalized PI-LC-X via ^{1}H NMR analysis..3
S3. The characterization of functionalized PI-LC-X via FTIR and Raman spectra................................4
S4. TGA measurement...6
S5. DSC measurement...7
S7. Tensile measurement..10
S8. Structural integrity assessment of PI-LC-30 via ^{1}H NMR analysis after three recycling cycles.....11
S9. Table comparison..12

S1. Photographs demonstrating the PI-LC-10 and control sample soaked in toluene

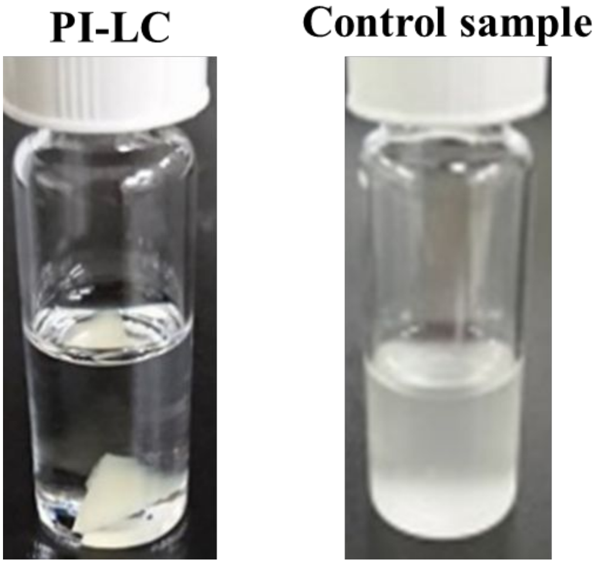

Fig. S1 Photographs showing the resultant PI-LC with and without (control sample) the involvement of initiator during the reaction, immersed in toluene to illustrate the impact of the initiator.

S2. The characterization of functionalized PI-LC-X via ¹H NMR analysis

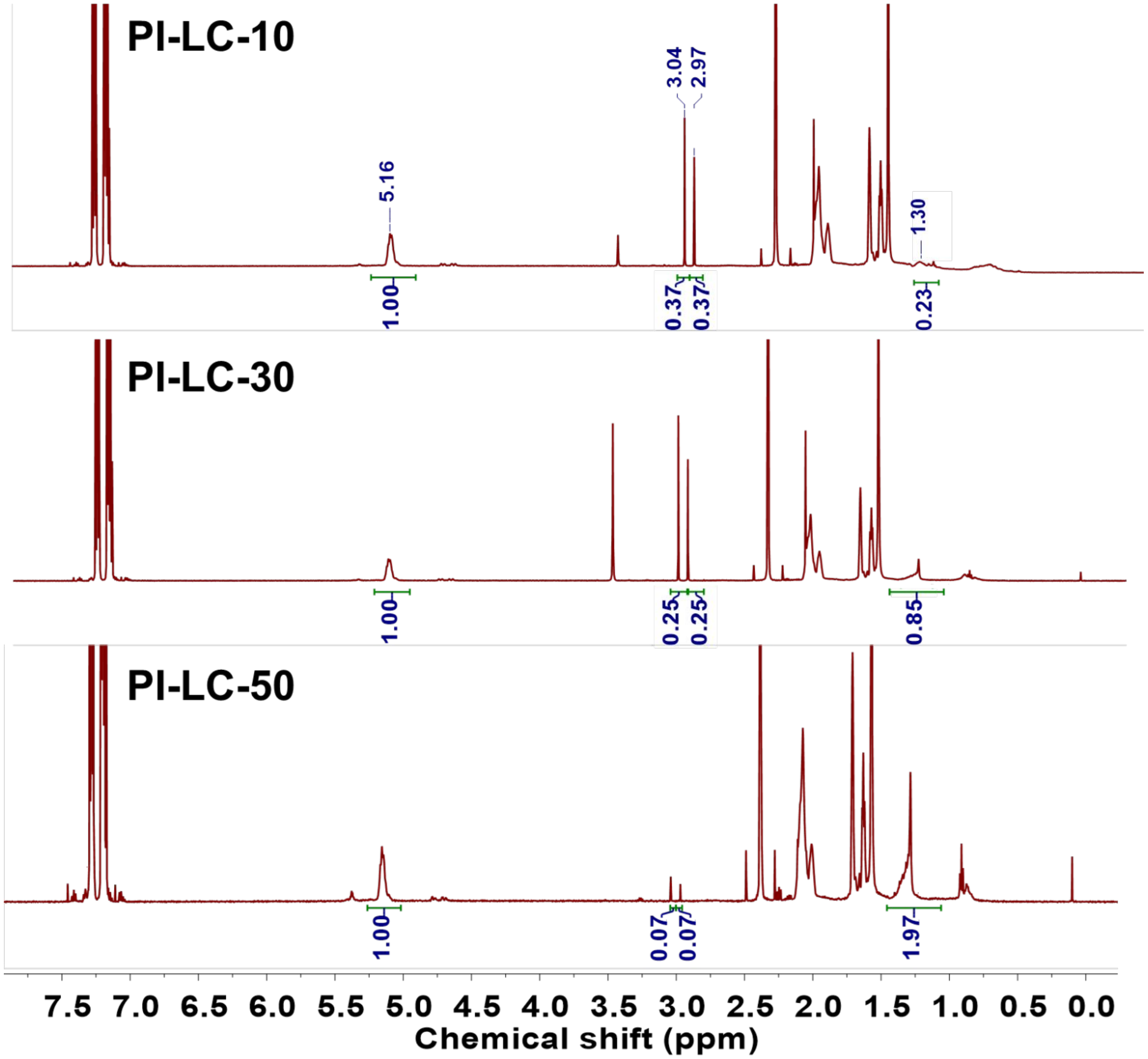

Fig. S2 The assignments and ¹H NMR spectra (400 MHz) of functionalized-PI-LC-X with variations in the number of LC attached to the PI backbone consisting of X = 10, 30, and 50 in $CDCl_3$_d.

S3. The characterization of functionalized PI-LC-X via FTIR and Raman spectra

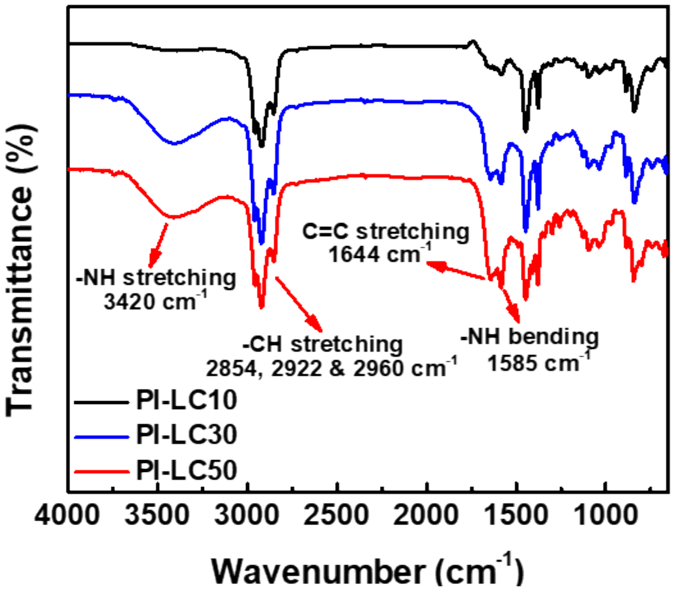

Fig. S3 FTIR analysis result of crosslinked-PI-LC-X with variations in the number of LC attached to the PI backbone consisting of X = 10, 30, and 50.

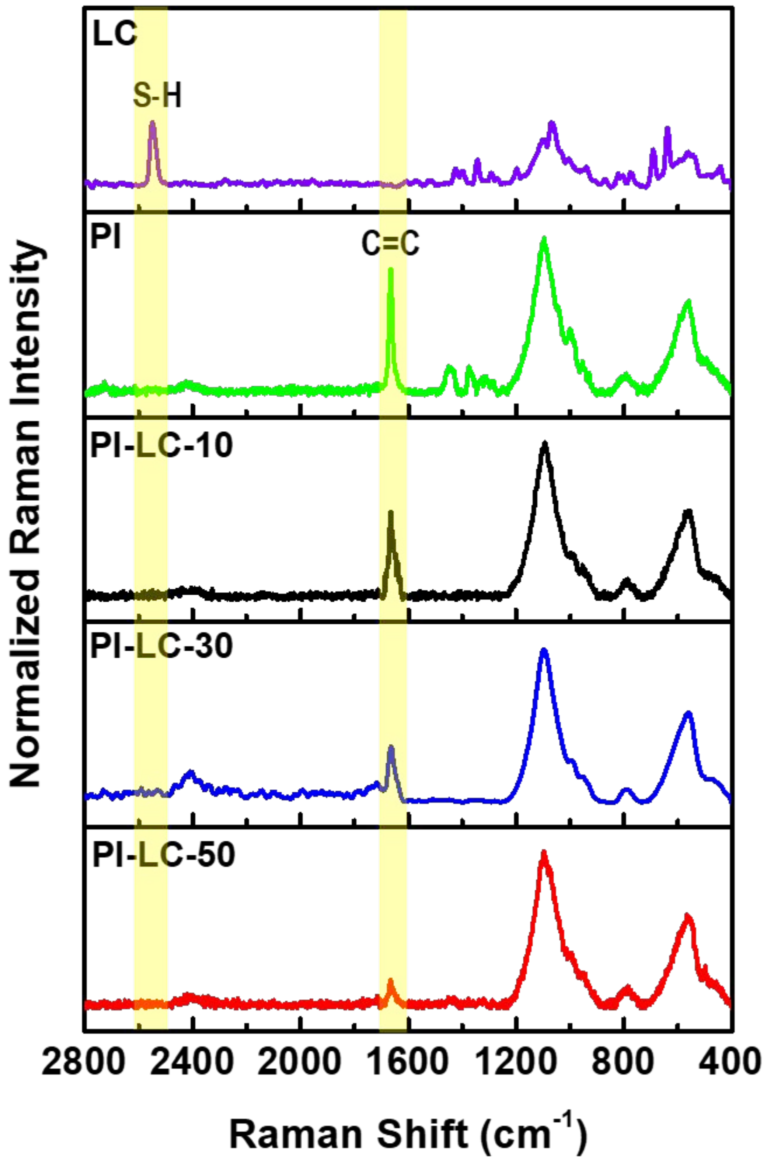

Fig. S4 Raman spectroscopy analysis of PI, LC, and functionalized PI-LC-X, revealed key spectral changes indicating successful free radical-mediated thiol-ene reaction characterized by disappearance of the peak S-H (~2551 cm^{-1}) and significant reduction of C=C (1664 cm^{-1}) across the PI-LC-X series.

S4. TGA measurement

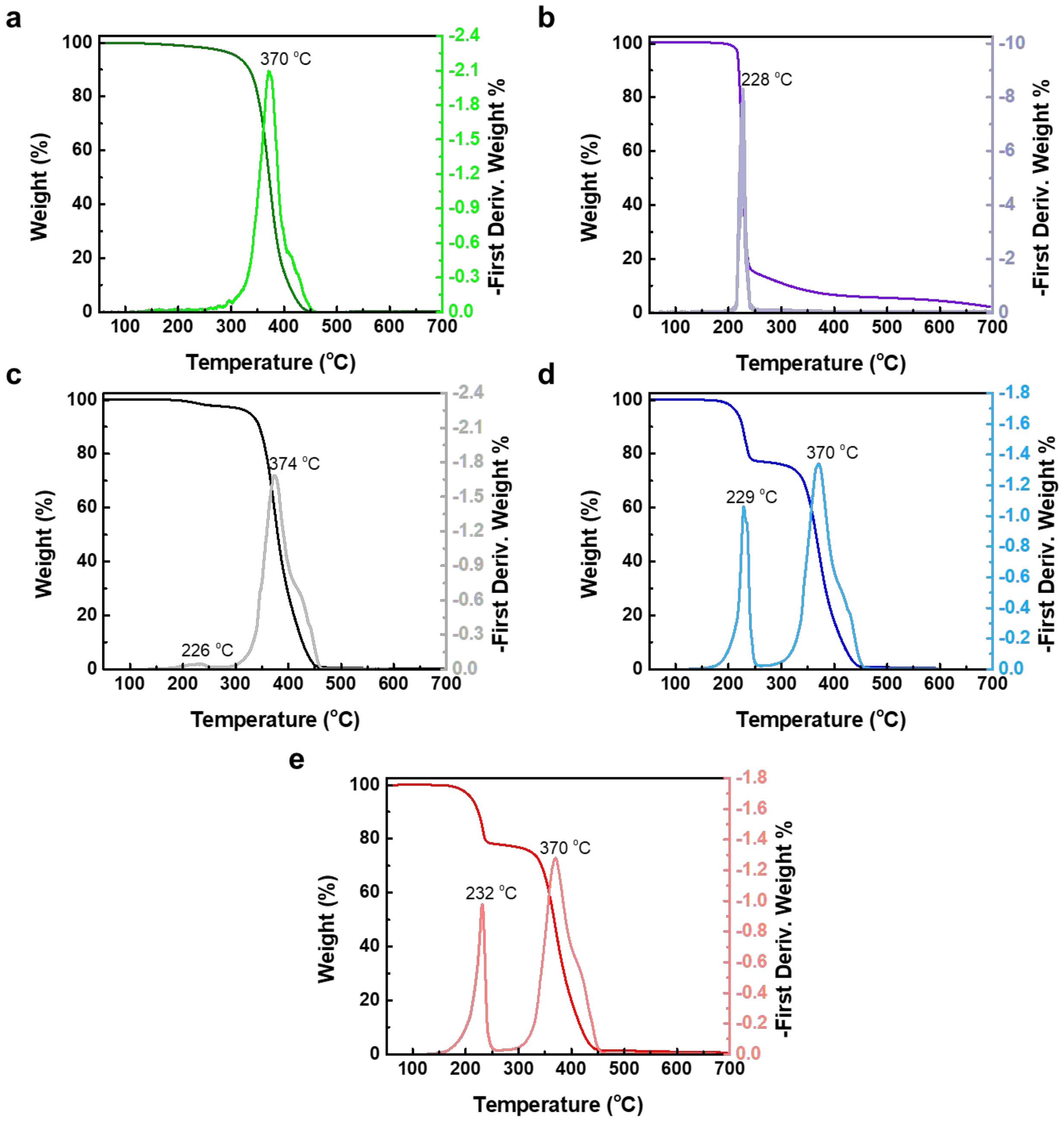

Fig. S5 TGA and first derivative thermogravimetric analysis (DTG) curves for (a) LC, (b) PI, (c) PI-LC-10, (d) PI-LC-30, and (e) PI-LC-50, highlighting the main decomposition temperatures corresponding to each component's degradation at distinct weight-loss stages.

S5. DSC measurement

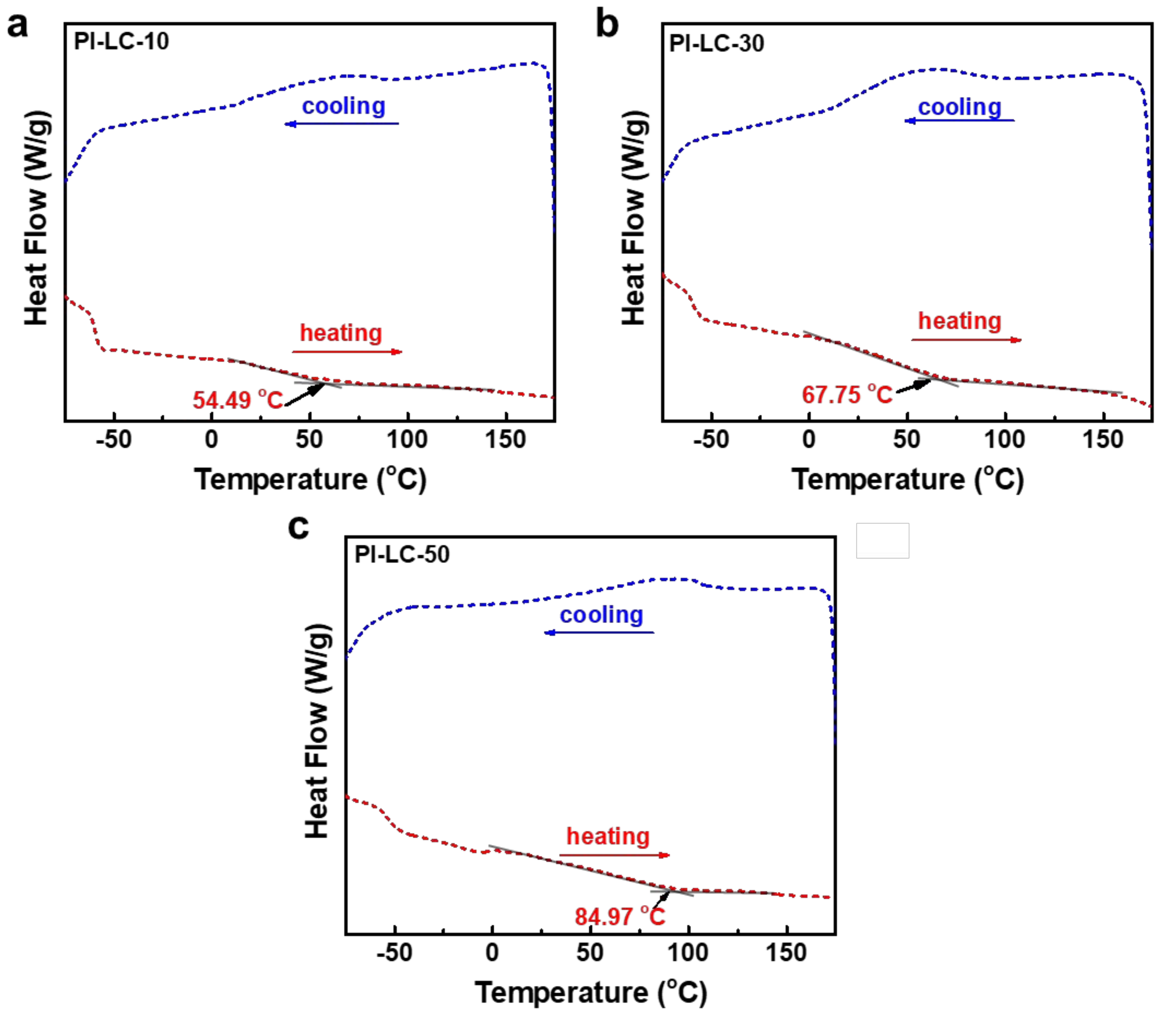

Fig. S6 DSC analysis with operating conditions in the range of -75 – 175℃ of (a) PI-LC-10, (b) PI-LC-30, and (c) PI-LC-50**.**

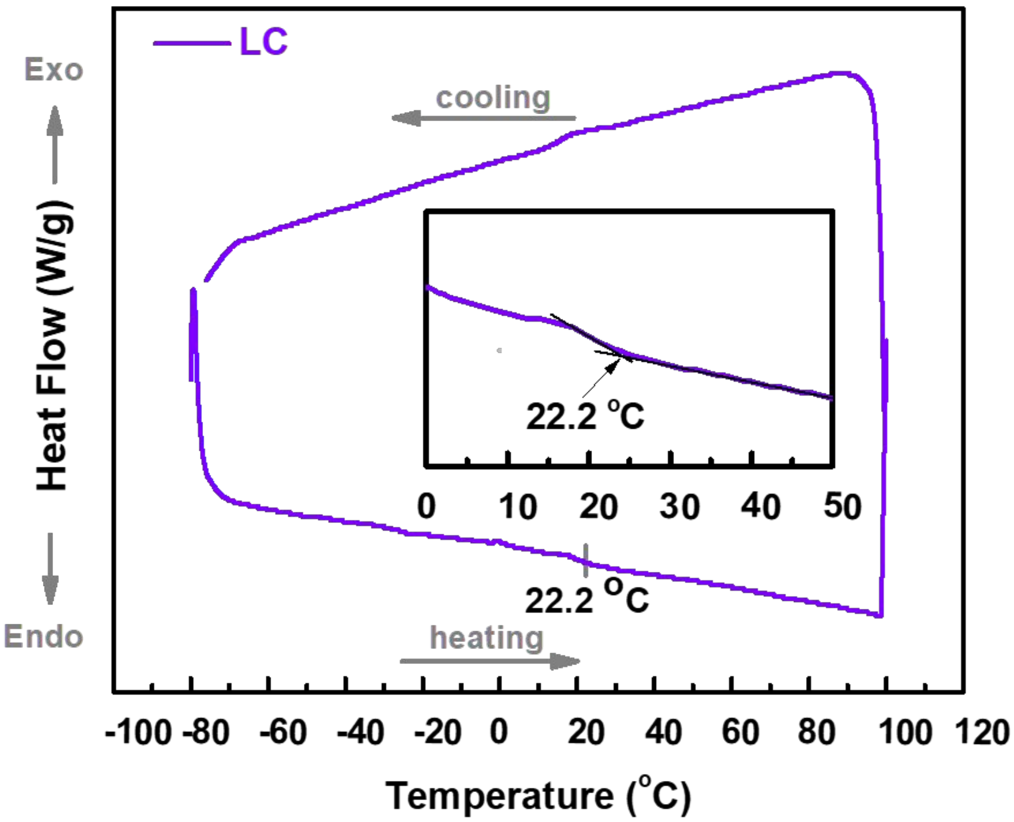

Fig. S7 DSC trace from the second heating cycle of pristine LC under operating conditions from -75 to 100°C, revealing a minor transition temperature. Inset: Enlargement of the DSC trace in the 0–50 °C range, showing two linear baselines before and after the step change, with their tangents intersecting to define the LC transition temperature.

S6. Observation thin film functionalized PI-LC-X using optical microscope

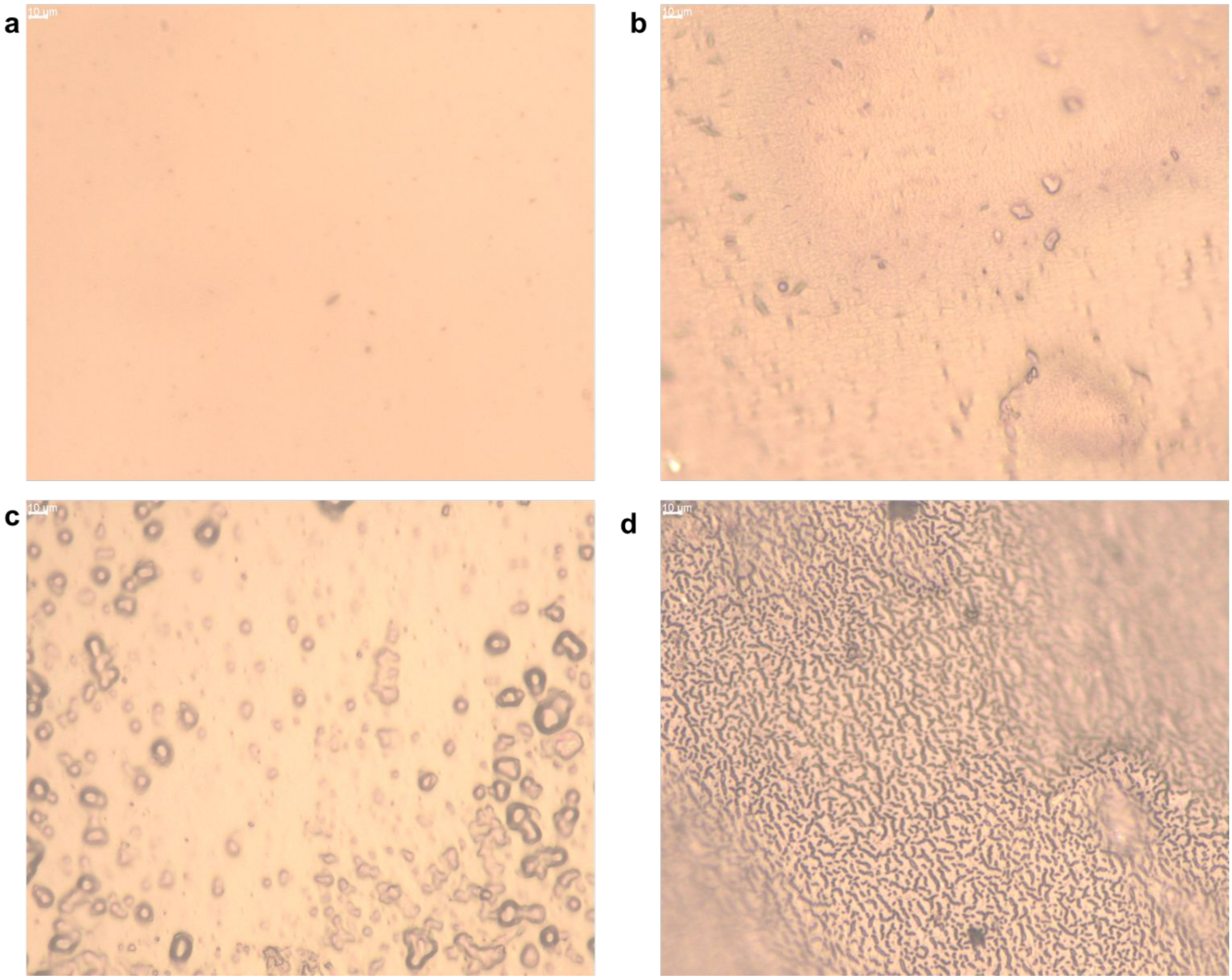

Fig. S8 Optical microscopy images (10 μm scale) of (a) pristine PI and PI-LC-X thin films with varying LC content of (b) PI-LC-10, (c) PI-LC-30, and (d) PI-LC-50. The surface morphologies of the PI and PI-LC-X thin films were observed at 50x magnification.

S7. Tensile measurement

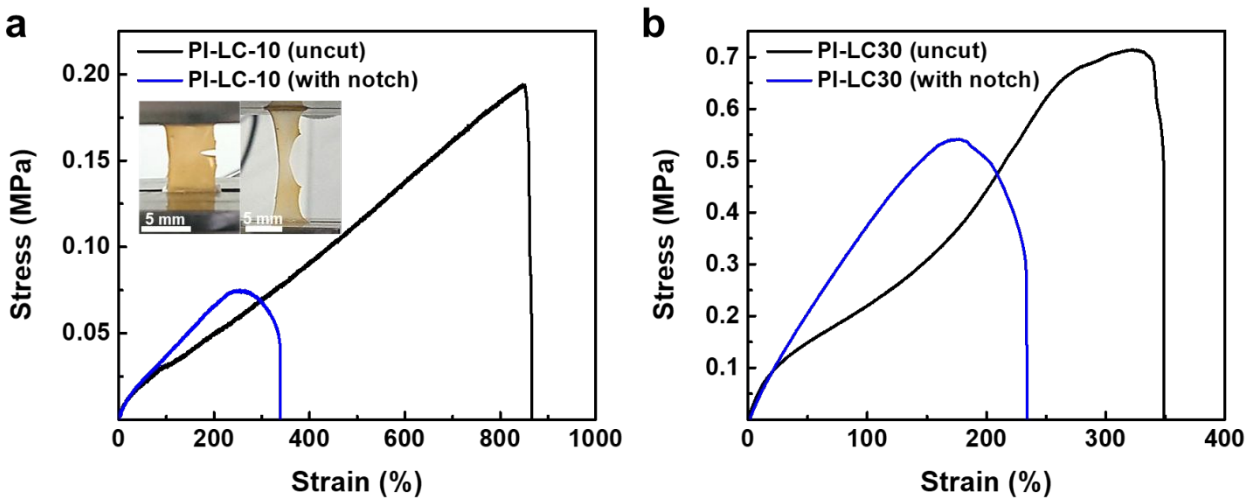

Fig. S9 Stress-strain curve results of the (a) PI-LC-10 and (b) PI-LC-30 films under notch conditions stretched to break.

S8. Structural integrity assessment of PI-LC-30 via ^{1}H NMR analysis after three recycling cycles

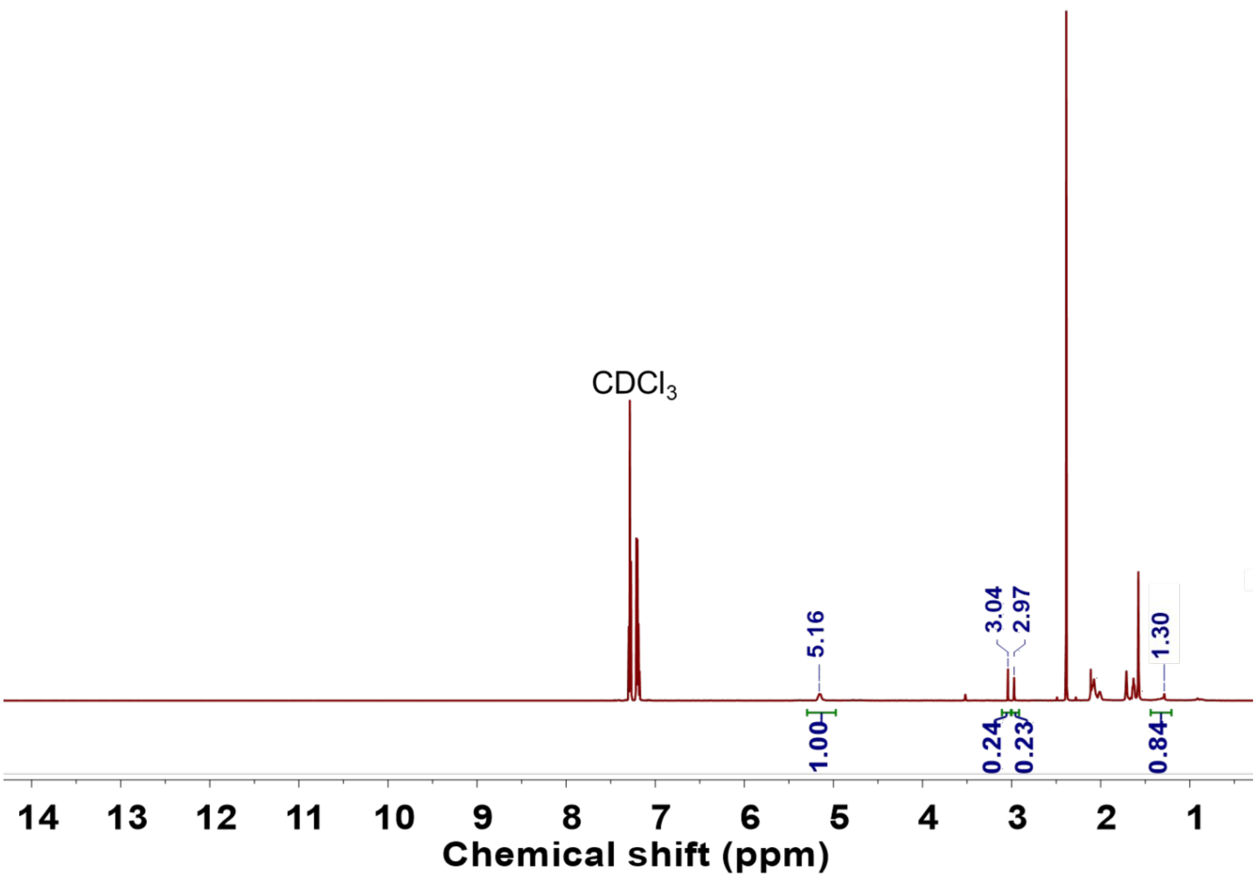

Fig. S10 ^{1}H NMR spectrum (400 MHz, $CDCl_3$_d) of PI-LC-30 after three recycling cycles. ^{1}H NMR overlays confirm chemical integrity with methylene signals adjacent to the thioether linkage (~3 ppm) and PI-bound LC (~1.3 ppm) unchanged. Absence of broad downfield signals (7–9 ppm region typical for $–NH_3^+$) confirms complete deprotonation.

S9. Table comparison

Table S1. Area integral calculations of the C=C peak intensity at 1664 cm^{-1} from Raman spectroscopy, confirming the high efficiency of the functionalization reaction.

Sample	Integral area peak C=C	% C=C[a]
LC	-	0
PI	19.08	100
PI-LC-10	17.22	90.25
PI-LC-30	14.06	73.69
PI-LC-50	9.96	52.20

[a]The percentage decrease in C=C levels was calculated as the ratio of the PI-LC-X peak intensity to that of PI, multiplied by 100%.

Table S2. Summary of mechanical properties (maximum tensile strength, maximum strain, and toughness) of vulcanized PI and PI-LC-X thin films with varying LC content.

Sample	Maximum stress (MPa)	Maximum strain (%)	Toughness (MJ m^{-3})
Vulcanized PI	0.19	193	0.22
PI-LC-10	0.20	865	0.87
PI-LC-30	0.71	348	1.39
PI-LC-50	0.85	266	1.42

Table S3. Summary of mechanical properties, including maximum tensile strength, maximum strain, and toughness, together with their corresponding relative standard deviations, for PI-LC-30 before and after three recycling cycles (measured at a stretching speed of 50 mm min^{-1}).

Condition	Maximum stress		Maximum strain		Toughness	
	Measured (MPa)	Relative standard deviation (%)	Measured (%)	Relative standard deviation (%)	Measured (MJ m^{-3})	Relative standard deviation (%)
Original	0.71	0	348	0	1.39	0
1st recycle	0.73	2.82	347	0.29	1.26	9.35
2nd recycle	0.77	8.45	371	6.61	1.63	17.27
3rd recycle	0.66	7.04	344	1.15	1.40	0.72
